# Interventions to increase the consumption of water among children: A systematic review and meta‐analysis

**DOI:** 10.1111/obr.13015

**Published:** 2020-03-13

**Authors:** Carmen B. Franse, Mirte Boelens, Lisa R. Fries, Florence Constant, Amy van Grieken, Hein Raat

**Affiliations:** ^1^ Department of Public Health Erasmus University Medical Center Rotterdam The Netherlands; ^2^ Nestlé Research Lausanne Switzerland; ^3^ Nestlé Waters MT Issy‐les‐Moulineaux France

**Keywords:** children, clinical trial, meta‐analysis, water

## Abstract

The aim of this study was to conduct a systematic review and meta‐analysis on the effectiveness of interventions to increase children's water consumption. A systematic literature search was conducted in seven electronic databases. Studies published in English before 18 February 2019 that evaluated any type of intervention that measured change in water consumption among children aged 2 to 12 years by applying any type of design were included. Of the 47 interventions included in the systematic review, 24 reported a statistically significant increase in water consumption. Twenty‐four interventions (17 randomized controlled trials and seven studies with other controlled designs) were included in the meta‐analysis. On average, children in intervention groups consumed 29 mL/d (confidence interval [CI] = 13–46 mL/d) more water than did children in control groups. This effect was larger in eight interventions focused specifically on diet (MD = 73 mL/d, CI = 20–126 mL/d) than in 16 interventions focused also on other lifestyle factors (MD = 15 mL/d, CI = 1–29 mL/d). Significant subgroup differences were also found by study setting and socioecological level targeted but not by children's age group, intervention strategy, or study design. In conclusion, there is evidence that, on average, lifestyle interventions can lead to small increases in children's daily water consumption. More research is needed to further understand the specific intervention elements that have the greatest effect.

## 1 INTRODUCTION

Water is a healthy alternative to sugar‐sweetened beverages (SSBs), of which high consumption has been associated with weight gain^1^, ^2^, ^3^ and tooth decay^4^, ^5^ in both children and adults. Guidelines therefore recommend introducing plain water when children are 6 months old and that it should be the principal source of hydration for children older than 1 year.^4^, ^5^, ^6^ In addition, the consumption of cow's milk for children older than 1 year is also recommended, because milk can contribute nutrients to childrens diet.^6^ Evidence from longitudinal studies suggest a weight‐reducing effect when consuming water instead of SSBs in children and adolescents^7^ as well as in adults.^8^ Some controlled trials have also found that promoting water consumption among children reduces weight gain.^9^, ^10^ Different mechanisms might underlie these findings. The total amount of calories consumed may be reduced as water contains no calories, whereas SSBs do.^11^, ^12^ Another mechanism supported by Varsamis et al may be that consuming SSBs is linked to elevated glucose responses and sustained elevation in plasma insulin during a day of prolonged sitting,^13^ which could lead to higher calorie intake in subsequent meals. A review by Daniels and Popkin suggested that the consumption of water instead of SSBs during or before meal times might reduce the energy intake during the meal.^14^

Choosing to drink water as the main beverage is a habit that is likely formed in childhood.^15^, ^16^ The family environment is viewed as the principal place where dietary habits are shaped, especially during early childhood.^17^ Parents create the food environment in the home and often act as the role models and gatekeepers for the dietary behaviours of their children.^18^, ^19^, ^20^ When children become older, the preschool and school environment can also influence the consumption behaviours of children.^21^, ^22^ Most interventions that target dietary and obesogenic behaviours have therefore been conducted in either the home or school environments; the latter, in particular, have received a lot of attention.^23^, ^24^ Some of these lifestyle interventions focus specifically on changing children's diet and the consumption of specific foods or beverages^25^, ^26^ or multiple types of foods or beverages.^27^ Other interventions focus on changing both dietary behaviour and other obesogenic lifestyle behaviours such as physical activity and sedentary behavior.^28^, ^29^

In a previous systematic review by our team, we identified potentially modifiable factors that were associated with children's water consumption; these factors were the child's self‐efficacy, parental self‐efficacy, and parental restrictive and encouraging feeding practices.^30^ By targeting such factors, lifestyle interventions may be able to promote the water intake among children. A positive effect of such lifestyle interventions has also been found for related outcomes such as the reduction of SSB consumption^21^, ^22^, ^31^ and reduction of weight gain.^22^, ^32^, ^33^ Although limited, recent evidence provides some indication that water consumption among children may indeed be promoted by interventions.^24^, ^34^ A systematic review by Cradock et al on interventions to increase drinking water access and consumption in children younger than 5 years found that 12 of the 18 studies that measured children's water consumption reported positive effects on water intake.^34^ A meta‐analysis on the effectiveness of lifestyle (diet with/without other obesogenic lifestyle behaviours) interventions on SSB and water consumption among children and adults by Vargas‐Garcia et al only included seven studies that targeted children and found an increase of 67 mL/d in children's water consumption.^24^ However, to date, no comprehensive and rigorous evidence exists on the effectiveness of interventions to promote water consumption among children of preschool and primary‐school age. The aim of this study was therefore to conduct a systematic review and meta‐analysis on the effectiveness of interventions to increase the consumption of water among children aged 2 to 12 years. We focused on children older than 2 years because patterns of and recommendations for beverage intake among children aged 0 to 2 years change substantially for water, breastmilk, cow's milk, and juice.^35^

## 2 METHODS

### 2.1 Search strategy

A systematic literature search for relevant studies was conducted in seven electronic databases: Embase, Medline Ovid, Web of Science, Cochrane, PsycINFO Ovid, CINAHL EBSCOhost, and Google Scholar on 11 May 2018.^30^ This search was updated in all seven electronic databases on 18 February 2019. A combination of key words was used in the search: (water or beverage* or drink* or related key words) and (child* or infant* or toddler* or related key words) and (intervention* or trial* or strateg* or effect* or promot* or related key words). The search strategy was adapted to each database. The full search strategies are presented in the Supporting Information. In addition to the database search, references of relevant articles were screened for other studies. The systematic review protocol for this study was registered in the PROSPERO registry under registration number CRD42019124808 on 18 April 2019.

### 2.2 Selection process

The duplicates of records retrieved in the search were removed. Subsequently, two independent reviewers (C.F., and L.W. or M.B.) performed title and abstract screening of the remaining records in order to identify studies that met inclusion criteria. Then, copies of full‐text articles were ordered for all remaining studies, and full‐text screening was performed by two independent reviewers (C.F., and L.W. or M.B.). At both stages, disagreements that arose were discussed between them and, if necessary, resolved by consultation with a senior reviewer (H.R.).

### 2.3 Inclusion and exclusion criteria

We applied specific inclusion criteria in the selection process. (a) We included participants with mean age between 2 and 12 years at baseline. (b) For the systematic review, we included any type of study design that allowed us to study the effectiveness of an intervention to increase the consumption of water among children, such as randomized controlled trials (RCTs), non‐RCTs, and other controlled and noncontrolled quasi‐experimental designs. For the meta‐analysis, we studied the mean difference between control group and intervention group in millilitres of water per day. Therefore, we could only include controlled studies that measured, within a specified time period, water consumption amount (millilitres, litres, grams, ounces, cups, glasses, and servings) and/or frequency (consumption occasions, consumption frequency, and consumption times). (c) We included any type of intervention strategy that aimed to promote water consumption among children. For studies that had a control group, the control group was defined as children who were not exposed to the intervention designed to promote water consumption. (d) We included the following categories of water: tap water, bottled drinking water, unflavoured sparkling water, flavoured water (nonsweetened), or any other source of safe drinking water. (e) We included studies that were published in an English‐language peer‐reviewed journal anytime up to 18 February 2019.

The main exclusion criteria that were applied during the selection process were as follows: (a) studies that only included participants from clinical populations (eg, obesity, malnutrition, and gastroenteritis), as we focused on the general population; (b) studies with data of less than 10 participants; and (3) studies that did not use human subjects. When more than one article was published on the same data set, the article with the longest follow‐up period was used. Pilot studies, five in total,^36^, ^37^, ^38^, ^39^, ^40^ were included when a full trial of the intervention was not available.

### 2.4 Data extraction

After discussion and consensus among the study team, a standardized data extraction form was developed. This form was used to extract data from the studies by one researcher (C.F.). The information that was extracted included author, year and country of study, study design and name, intervention content (setting, strategy, socioecological level targeted, focus, frequency, and theory used), control condition, length of intervention and follow‐up time, population age and characteristics, number of participants in intervention and control groups and number of clusters (if applicable), how water consumption was measured, participation and retention rate, and outcome data: effect of the intervention on water consumption among children. Key data (intervention content and outcome data) were checked by a second researcher (M.B.). If available, published protocol papers were obtained and used during data extraction.

For the purpose of the meta‐analysis, continuous data were extracted either as mean with standard deviation or as adjusted mean difference with standard error. When studies had multiple follow‐up time points, the time point with the longest follow‐up time was chosen as suggested in the Cochrane Handbook.^41^ For two studies that had multiple intervention arms with different intervention elements,^42^, ^43^ we used the intervention arm with all intervention elements combined. For one study that had two slightly different intervention arms,^44^ the average of the two intervention arms was used as recommended in the Cochrane Handbook.^41^ For specific choices for each paper, see Table S1. When data were missing, the authors were contacted to obtain the missing data.

### 2.5 Risk of bias assessment

The risk of bias was assessed independently by two reviewers (C.F. and M.B.). For RCTs, the Cochrane Collaboration's tool for assessing risk of bias was used.^45^ This tool assesses bias in random sequence generation; in allocation concealment; in blinding of participants, personnel, and outcome assessors; because of incomplete outcome data; because of selective reporting; and because of other reasons. For each domain of bias, the study was categorized as having “low” or “high” risk of bias. When it was not possible to determine the risk of bias for a certain bias domain because of missing information in the article, the domain was coded as “unclear.” The most serious rating across these bias domains determined the overall risk of bias; eg, if a study was categorized as having a “low” risk of bias in five domains but a “high” risk of bias in one domain, the overall risk of bias was high. For other designs, the Risk Of Bias In Nonrandomized Studies of Interventions (ROBINS‐I) was used.^46^ The ROBINS‐I tool assesses bias because of confounding, in the selection of participants into study, in classification of exposures, because of departures from intended exposures, because of missing data, in measurement of outcomes, and in selection of the reported result. For each domain, the study was categorized as having “low,” “moderate,” “serious,” or “critical” risk of bias. For example, for the “bias due to confounding” domain, we assessed whether the study corrected for confounding variables, such as the child's sex and age. When it was not possible to define the risk of bias for a specific bias domain because of missing information, the domain was coded as “no information.” Again, the most serious rating across bias domains defined the overall risk of bias. When there were discrepancies in the judgement of bias between the two reviewers, these were resolved through discussion.

### 2.6 Analysis

For the qualitative synthesis, we calculated the number of interventions that found a (statistically significant) positive effect on water consumption among children out of the total number of interventions included in the systematic review. We conducted a meta‐analysis only with the subset of interventions with a controlled study design and appropriate outcome data available. A random‐effects meta‐analysis was conducted to account for the between‐study variance using the mean difference in millilitres of water consumption at follow‐up between the intervention group and control group and the standard error of this difference. The overall mean difference and 95% confidence interval (CI) across all studies were estimated, and forest plots were created that graphically display these results. The meta‐analysis was conducted in Review Manager (version 5.3, Cochrane Library). The Cochrane Handbook was used for guidance regarding missing data and combining of data,^41^ and the Cochrane calculator was used for making calculations. Results from the most adjusted models were used, wherever available. If the mean difference in water consumption between the control group and intervention group at follow‐up was not reported, the mean water consumption at follow‐up was extracted separately for the intervention group and control group, and the mean difference between intervention and control group was calculated. Using follow‐up scores instead of change from baseline to follow‐up scores is suggested to generate more conservative results in meta‐analyses.^33^, ^47^

If in the paper water consumption was reported in a different quantity than in millilitres or within a different time period than 1 day, consumption was recalculated to millilitres of water per day. If the size of the portions was not reported in the paper, we used a portion size of 225 mL per drink or consumption occasion. This was chosen because among the included papers that reported portion sizes, these varied between 200 and 250 mL.^9^, ^48^, ^49^, ^50^, ^51^ If CIs were presented instead of standard errors or standard deviations, these were calculated with the Cochrane calculator. When data were presented stratified by subgroups, such as by sex, subgroups were combined. If standard deviation at follow‐up was missing but standard deviation at baseline was available, this was used. For specific calculations for each paper, see Table S1.

The *I* ^2^ test was used to assess heterogeneity across studies; above 25% is considered low variance between studies, above 50% is considered moderate variance, and above 75% is considered high variance.^52^ As specified in our protocol, subgroup analyses were performed with (a) potential moderators: (1) type of intervention; focus (diet vs diet + other lifestyle factors) and strategy (education only vs other strategies + education vs only other strategies), (2) socioecological level targeted by the intervention (individual level [ie, the child] vs interpersonal level [ie, the parent or peer] vs environmental level vs all levels), (3) children's age group targeted (2–5 vs 6–12 y), and (4) setting (school vs nonschool vs both school and nonschool) and (b) with type of study design (RCT vs other controlled designs). A sensitivity analysis was performed by reestimating the overall effect in forest plots with papers using the mean water consumption and standard deviation in intervention group and control group at follow‐up. A funnel plot was created of all studies and inspected visually.

## 3 RESULTS

### 3.1 Study selection

**Figure 1:**
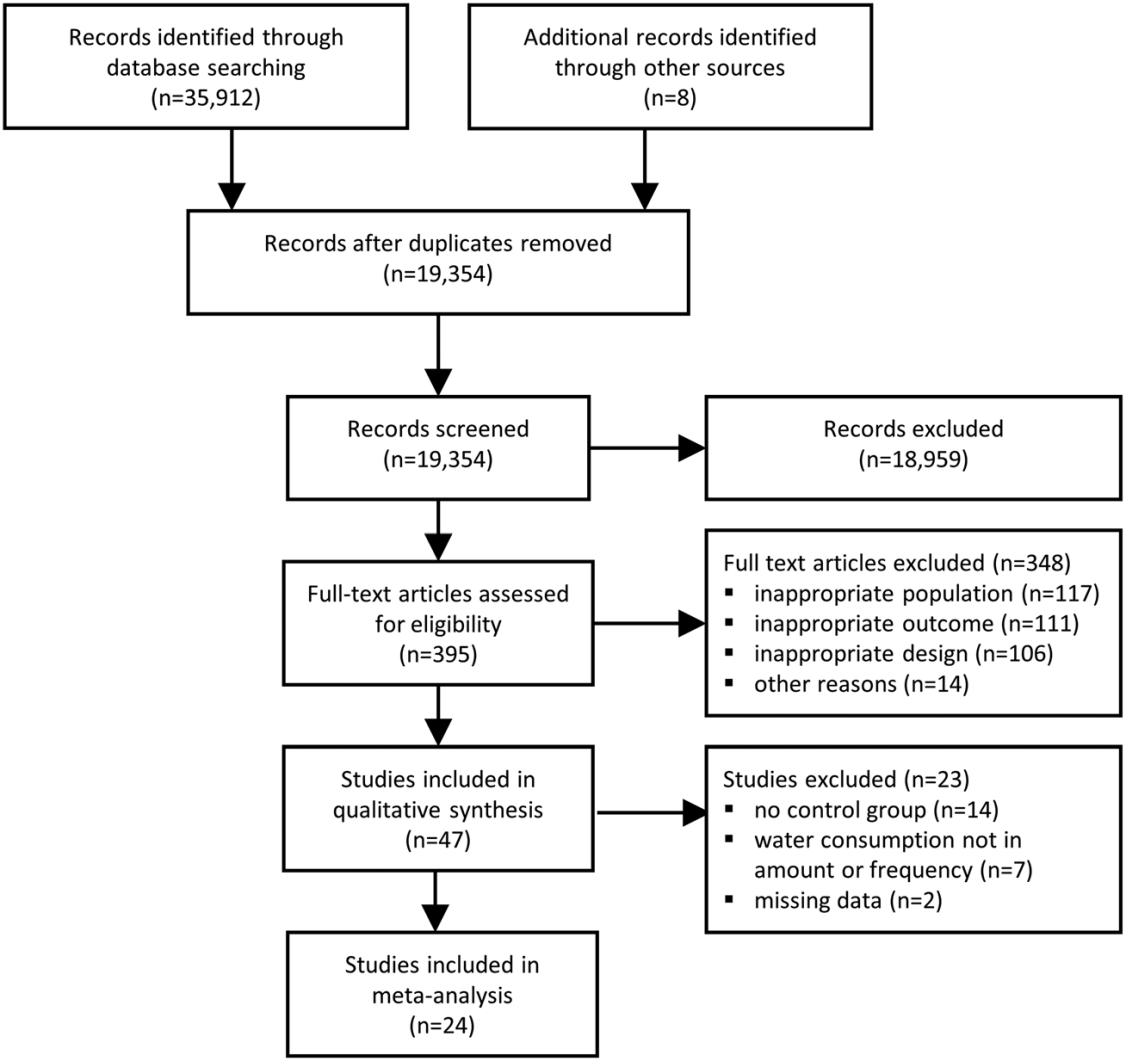
Flow chart for the selection of reviewed studies

The inclusion and exclusion of articles are described using the Preferred Reporting Items for Systematic Reviews and Meta‐Analyses (PRISMA)^53^ flow chart (Figure 1). A total of 35 912 records were identified through the database search. After removal of duplicates, a total of 19 346 records remained. After all rounds of screening, 39 articles were identified and included. Eight additional studies were identified by hand searching references of included articles and other relevant articles, resulting in 47 articles that met the inclusion criteria and were included in the systematic review.^9^, ^36^, ^37^, ^38^, ^39^, ^40^, ^42^, ^43^, ^44^, ^48^, ^49^, ^50^, ^51^, ^54^, ^55^, ^56^, ^57^, ^58^, ^59^, ^60^, ^61^, ^62^, ^63^, ^64^, ^65^, ^66^, ^67^, ^68^, ^69^, ^70^, ^71^, ^72^, ^73^, ^74^, ^75^, ^76^, ^77^, ^78^, ^79^, ^80^, ^81^, ^82^, ^83^, ^84^, ^85^, ^86^, ^87^ Of these 47 studies, 24 studies could be included in the meta‐analysis because they had a controlled design and measured water consumption in an amount or frequency.^9^, ^40^, ^42^, ^43^, ^48^, ^49^, ^50^, ^51^, ^54^, ^60^, ^61^, ^63^, ^70^, ^72^, ^73^, ^74^, ^75^, ^77^, ^80^, ^81^, ^82^, ^83^, ^84^, ^85^

### 3.2 Study characteristics

The overall characteristics of the studies that were included in the systematic review are shown in Table 1; specific details of each study are shown in Table S2. Of the 47 studies, the majority were based in the United States (24/47)^37^, ^38^, ^39^, ^43^, ^48^, ^50^, ^54^, ^55^, ^56^, ^57^, ^62^, ^64^, ^66^, ^67^, ^68^, ^70^, ^72^, ^73^, ^75^, ^76^, ^78^, ^80^, ^81^, ^87^ or in Europe (14/47).^9^, ^40^, ^42^, ^44^, ^51^, ^58^, ^59^, ^60^, ^61^, ^74^, ^77^, ^83^, ^84^, ^85^ All studies were published after 2000, and most (40/47)^36^, ^37^, ^39^, ^40^, ^42^, ^43^, ^44^, ^48^, ^49^, ^50^, ^54^, ^55^, ^56^, ^57^, ^58^, ^59^, ^60^, ^61^, ^62^, ^63^, ^64^, ^65^, ^66^, ^67^, ^68^, ^69^, ^70^, ^72^, ^74^, ^75^, ^76^, ^77^, ^78^, ^79^, ^80^, ^81^, ^83^, ^84^, ^86^, ^87^ were published in or after 2010. Most studies were RCTs (24/47)^40^, ^43^, ^44^, ^48^, ^50^, ^51^, ^54^, ^57^, ^59^, ^61^, ^63^, ^66^, ^69^, ^70^, ^74^, ^75^, ^76^, ^77^, ^80^, ^81^, ^83^, ^84^, ^85^, ^86^; other designs were non‐RCTs (9/47),^9^, ^39^, ^42^, ^60^, ^62^, ^67^, ^72^, ^73^, ^82^ repeated cross‐sectional controlled (1/47),^49^ or noncontrolled quasi‐experimental study designs (13/47).^36^, ^37^, ^38^, ^55^, ^56^, ^58^, ^64^, ^65^, ^68^, ^71^, ^78^, ^79^, ^87^

**Table 1:** Characteristics of the studies included in the systematic review (*N* = 47)

Characteristics	n (%) studies	
Study location^a^		
The United States	24	(51)
Europe	14	(30)
Australia/New Zealand	4	(9)
Mexico/South America	4	(9)
Middle East	2	(4)
Year published		
≥ 2010	40	(85)
2000–2009	7	(15)
Design		
Randomized controlled trial	24	(51)
Nonrandomized controlled trial	9	(19)
Repeated cross‐sectional controlled	1	(2)
Noncontrolled quasi‐experimental study	13	(28)
Number of participants^b^		
< 300	14	(30)
300–999	22	(47)
≥ 1000	8	(17)
Mean age children		
Preschool aged (2–5 y)	16	(34)
School aged (6–12 y)	31	(66)
Intervention setting		
School/preschool	28	(60)
Community	7	(15)
Home	4	(9)
Multiple	8	(17)
Length of intervention		
≤ 6 mo	25	(53)
> 6 mo	22	(47)
Focus of intervention		
Diet and other lifestyle factors	23	(49)
Diet	11	(23)
Beverages	13	(28)
Theory‐based intervention		
Theory reported	40	(85)
No theory reported	7	(15)
Socioecological level targeted (multiple possible)		
Individual level (child)	37	(79)
Interpersonal level (parent/peer)	28	(60)
Environmental level (school/home/community)	30	(64)
Intervention strategy (multiple possible)		
Education	33	(70)
Restructuring environment	21	(45)
Social marketing	13	(28)
Computer/online program	4	(9)
Peer influence	3	(6)
Measurement instrument of water consumption		
Food frequency questionnaire	26	(55)
24‐h recall	11	(23)
Prospective dietary records	4	(9)
Observation	6	(13)
Outcome water consumption		
Volume consumed	14	(30)
Glasses/servings consumed	11	(23)
Consumption occasions	12	(26)
Proportion children that consumed water	10	(21)

Total is 48, because one study was located in Mexico and the United States.

For three studies, the number of participants was not reported, and only the number of schools/programs was reported.

Sixteen interventions targeted preschool‐aged children,^37^, ^42^, ^43^, ^49^, ^58^, ^59^, ^60^, ^61^, ^68^, ^69^, ^73^, ^74^, ^75^, ^77^, ^79^, ^85^ and the other interventions (31/47)^9^, ^36^, ^38^, ^39^, ^40^, ^44^, ^48^, ^50^, ^51^, ^54^, ^55^, ^56^, ^57^, ^62^, ^63^, ^64^, ^65^, ^66^, ^67^, ^70^, ^71^, ^72^, ^76^, ^78^, ^80^, ^81^, ^82^, ^83^, ^84^, ^86^, ^87^ targeted school‐aged children. The majority of interventions were based in a school and/or preschool setting (28/47)^9^, ^36^, ^38^, ^39^, ^40^, ^48^, ^51^, ^55^, ^57^, ^58^, ^59^, ^62^, ^63^, ^65^, ^66^, ^69^, ^71^, ^72^, ^76^, ^77^, ^78^, ^80^, ^81^, ^82^, ^84^, ^85^, ^86^, ^87^; other settings were the community (7/47)^37^, ^56^, ^64^, ^67^, ^68^, ^70^, ^73^ and home (4/47),^42^, ^44^, ^54^, ^74^ and some interventions were based in multiple settings (8/47).^43^, ^49^, ^50^, ^60^, ^61^, ^75^, ^79^, ^83^ Around half of the interventions focused on changing children's diet and other lifestyle factors (23/47),^37^, ^38^, ^43^, ^44^, ^48^, ^49^, ^54^, ^60^, ^61^, ^62^, ^69^, ^70^, ^72^, ^73^, ^75^, ^77^, ^79^, ^80^, ^81^, ^82^, ^84^, ^86^, ^87^ while some interventions focused specifically on children's diet (11/47)^55^, ^56^, ^57^, ^58^, ^59^, ^64^, ^65^, ^67^, ^71^, ^74^, ^85^ or consumption of beverages (13/47).^9^, ^36^, ^39^, ^40^, ^42^, ^51^, ^62^, ^63^, ^66^, ^68^, ^76^, ^78^, ^83^ Most studies (40/47)^9^, ^36^, ^37^, ^38^, ^40^, ^42^, ^43^, ^44^, ^48^, ^49^, ^50^, ^54^, ^56^, ^57^, ^59^, ^60^, ^61^, ^62^, ^63^, ^64^, ^65^, ^66^, ^68^, ^69^, ^70^, ^71^, ^72^, ^73^, ^74^, ^75^, ^76^, ^77^, ^79^, ^80^, ^81^, ^83^, ^84^, ^85^, ^86^, ^87^ reported the use of theories for intervention development; only seven studies^39^, ^51^, ^55^, ^58^, ^67^, ^78^, ^82^ did not report any theory. The majority of studies targeted the individual socioecological level, ie, the child (37/47),^9^, ^36^, ^37^, ^38^, ^40^, ^42^, ^43^, ^44^, ^48^, ^50^, ^51^, ^54^, ^55^, ^56^, ^58^, ^59^, ^60^, ^61^, ^62^, ^63^, ^64^, ^65^, ^68^, ^69^, ^70^, ^71^, ^72^, ^77^, ^78^, ^79^, ^80^, ^82^, ^83^, ^84^, ^85^, ^86^, ^87^ and over half of the interventions targeted the interpersonal level, ie, parents or peers (28/47)^37^, ^40^, ^42^, ^43^, ^44^, ^49^, ^50^, ^56^, ^58^, ^59^, ^60^, ^61^, ^62^, ^63^, ^64^, ^67^, ^68^, ^69^, ^70^, ^73^, ^74^, ^75^, ^77^, ^79^, ^81^, ^83^, ^85^, ^87^ or the environmental level (30/47).^9^, ^39^, ^42^, ^43^, ^49^, ^50^, ^56^, ^57^, ^58^, ^60^, ^61^, ^62^, ^63^, ^65^, ^66^, ^67^, ^68^, ^69^, ^71^, ^75^, ^76^, ^77^, ^78^, ^79^, ^80^, ^81^, ^82^, ^83^, ^84^, ^86^ Education was used as a strategy in the majority of interventions (33/47)^9^, ^36^, ^37^, ^38^, ^42^, ^43^, ^48^, ^50^, ^51^, ^54^, ^55^, ^58^, ^59^, ^60^, ^61^, ^62^, ^64^, ^69^, ^70^, ^71^, ^72^, ^73^, ^74^, ^75^, ^77^, ^78^, ^79^, ^81^, ^82^, ^83^, ^84^, ^85^, ^87^; other strategies that were used were restructuring the environment (21/47),^9^, ^39^, ^42^, ^49^, ^50^, ^57^, ^58^, ^60^, ^62^, ^66^, ^67^, ^71^, ^75^, ^76^, ^77^, ^78^, ^80^, ^81^, ^82^, ^83^, ^84^ social marketing (13/47),^39^, ^44^, ^49^, ^61^, ^65^, ^67^, ^68^, ^71^, ^75^, ^76^, ^78^, ^80^, ^83^ computer/online programs (4/47),^42^, ^44^, ^54^, ^72^ and peer influence (3/47).^40^, ^42^, ^63^

The food frequency questionnaire (FFQ) was the most commonly used assessment tool (26/47 studies)^36^, ^40^, ^43^, ^48^, ^50^, ^58^, ^59^, ^60^, ^61^, ^63^, ^64^, ^67^, ^68^, ^69^, ^72^, ^74^, ^76^, ^77^, ^79^, ^80^, ^81^, ^82^, ^83^, ^84^, ^85^, ^87^ to measure water consumption. The most common outcome measure was water consumption in volume (14/47 studies)^36^, ^37^, ^42^, ^48^, ^54^, ^61^, ^65^, ^66^, ^75^, ^77^, ^80^, ^83^, ^84^, ^85^ such as millilitres, ounces, or cups.

### 3.3 Risk of bias

Risk of bias assessment of RCTs is reported in Table S3, and risk of bias of other designs is reported in Table S4. Among the 24 RCTs, overall risk of bias was classified as high in 19/24 studies, low in 1/24 studies, and unclear in 4/24 studies. On average, among the six bias domains, 51% domains were classified as “low bias,” 26% as “high bias,” and 22% as “unclear bias.” Among the studies with other designs, overall risk of bias was classified as serious in 21/23 studies and critical in 2/23 studies. On average, among the seven bias domains, 46% domains were classified as “low or moderate bias,” 33% as “serious bias,” 1% as “critical bias,” and 20% as “unclear bias or not applicable.” A large source of risk of bias was measurement of outcome for both RCTs and other designs, due to reliance on 1‐day 24‐hour recall or FFQ; 18/24 RCTs used this and 20/23 of studies with other designs. Retrospective recall for self‐reported or proxy‐reported fluid intake is considered to be unreliable, and repeated recalls and diaries are the most appropriate report‐based methods to assess fluid intake in children and adolescents.^88^ Risk of bias due to incomplete outcome/missing data was high in only 6/24 RCTs and unclear in 3/24 RCTs, but for studies with other designs, this was unclear in 5/23 studies, serious in 8/23 studies, and critical in 1/23 studies. Another frequent source of risk of bias for RCTs was not blinding of participants, personnel, and outcome assessors, which was high in 3/24 RCTs and unclear for 14/24 RCTs because it was not reported. However, blinding is not feasible for interventions that use education or restructuring of the environment. For studies with other designs, a large source of risk of bias was due to possible confounding, which was serious for 13/23 studies that did not correct for confounding variables such as sex, age, and baseline water consumption. Risk of bias in the other bias domains—random sequence generation, allocation concealment and selective outcome reporting for RCTs and selection of participants, classification of/departures from interventions, and selection of the reported result for other designs—was lower than that in the aforementioned domains.

### 3.4 Effectiveness of interventions on water consumption in children

Of the 47 studies included in our review, 24 reported statistically significant effects on children's water consumption (Table S2). Among studies that focused on diet and other lifestyle factors, 9/23 (39%) studies reported significant effects; among studies that focused on diet only, 6/11 (55%) studies reported significant effects; and among studies that focused on beverage consumption only, 9/13 (69%) studies reported significant effects. Among the interventions based at school, 16/28 (57%) reported significant effects; among the interventions in nonschool settings, 7/11 (64%) reported significant effects; and among interventions based at both school and nonschool settings, 1/8 (13%) reported significant effects. Among interventions that only targeted the individual socioecological level, 2/7 (29%) reported significant effects; among interventions that targeted the interpersonal level combined with the individual level, 6/10 (60%) reported significant effects; among interventions that targeted the environmental level only or combined with one other level, 10/17 (59%) reported significant effects; and among interventions that targeted all levels, 6/13 (46%) reported significant effects. Among the RCTs, 9/24 (38%) studies reported significant effects between intervention and control group; and among other designs, 15/23 (65%) studies reported significant effects.

### 3.5 Meta‐analysis

#### 3.5.1 Overall effects

**Figure 2:**
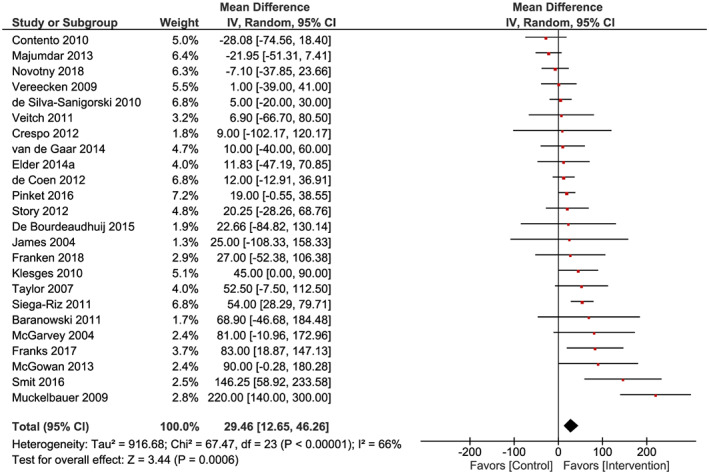
Random‐effects meta‐analysis of the mean difference in children's water consumption (in millilitre per day) between intervention and control groups (*n* = 24)

Results from the 24 studies included in the meta‐analysis show that the interventions increased water consumption among children (Figure 2). The mean difference between control and intervention groups was 29 mL/d (CI = 13; 46 mL/d, *N* = 32 206, *Z* = 3.36, *P* < .001). The studies were significantly heterogeneous (*χ* ^2^ = 67.47, df = 23, *P* < .001, *I* ^2^ = 66%).

#### 3.5.2 Effect by focus of intervention

There were 16 interventions that focused on diet and other lifestyle factors and eight interventions that focused on diet only (of which six focused only on beverage consumption). Interventions that focused on diet and other lifestyle factors had overall smaller mean differences between intervention and control groups (Table 2 and Figure S1: MD = 15 mL/d, CI = 1; 29 mL/d, *P* = .03, *I* ^2^ = 44%) than had studies that focused only on diet or beverages (MD = 73 mL/d, CI = 20; 126 mL/d, *P* = .007; *I* ^2^ = 78%). This difference was statistically significant (*P* = .04).

**Table 2:** Subgroup analyses using random‐effects models of the mean difference in childrens water consumption between intervention and control groups (n = 24)

	Number of studies	Estimate, mL/d	95% CI, mL/d	*P* value estimate	Heterogeneity (*I* ^2^), %	*P* value subgroup difference(s)
Focus of intervention						.04
Diet	8	72.80	19.51–126.09	.007	78	
Diet and other lifestyle factors	16	15.36	1.40–29.32	.03	44	
Intervention strategy						.33
Education only	8	16.59	−14.23 to 47.42	.29	54	
Other strategies with/without education	16	34.70	14.62–54.77	<.001	69	
Intervention setting						.002
School	13	32.99	6.03–59.95	.02	77	
Nonschool	5	64.70	33.67–95.74	<.001	0	
School and nonschool	6	5.48	−8.89 to 19.86	.45	0	
Socioecological level targeted						.004
Individual level only	4	−18.92	−42.90 to 5.06	.12	0	
Interpersonal—With/without individual level	6	54.87	13.85–95.88	.009	56	
Environmental—With/without one other level	7	41.77	4.47–79.08	.03	83	
All levels	7	18.65	4.95–32.35	.008	0	
Mean age of children						.16
2–5 y	10	15.56	1.49–29.63	.03	27	
6–12 y	14	40.12	9.26–70.99	.01	75	
Study design						.19
Randomized controlled trial	17	21.36	6.34–36.38	.005	42	
Other controlled design	7	56.81	6.46–107.15	.03	85	

Abbreviation: CI, confidence interval.

#### 3.5.3 Effect by intervention strategy

Eight interventions that only used education as an intervention strategy had an overall smaller mean difference between intervention and control groups (Table 2 and Figure S2: MD = 17 mL/d, CI = −14; 47 mL/d, *P* = .29, *I* ^2^ = 54%) than had 16 interventions that used other strategies such as restructuring the environment or social marketing with/without education (MD = 35 mL/d, CI = 15; 55 mL/d, *P* < .001, *I* ^2^ = 69%). This difference was not statistically significant (*P* = .33).

#### 3.5.4 Effect by intervention setting

There were 13 interventions that were in a school setting, five interventions that were based in a nonschool setting (community or home), and six interventions that were based in both a school and nonschool setting. Interventions based in both a school and nonschool setting had the smallest mean difference between intervention and control groups (Table 2 and Figure S3: MD = 5 mL/d, CI = −9; 20 mL/d, *P* = .96, *I* ^2^ = 0%), followed by interventions that were based in a school setting (MD = 33 mL/d, CI = 6; 60 mL/d, *P* = .02; *I* ^2^ = 77%) and interventions that were based in a nonschool setting (MD = 65 mL/d, CI = 34; 96 mL/d, *P* < .001; *I* ^2^ = 0%). Differences between these three groups were statistically significant (*P* = .002).

#### 3.5.5 Effect by socioecological level targeted

Four interventions that only targeted the individual level had the lowest and negative mean difference between intervention and control groups (Table 2 and Figure S4: MD = −19 mL/d, CI = −43; 5 mL/d, *P* = .12, *I* ^2^ = 0%), followed by seven interventions that targeted all levels (MD = 19 mL/d, CI = 5; 32 mL/d, *P* = .008, *I* ^2^ = 0%), seven interventions that targeted the environment with/without one other level (MD = 42 mL/d, CI = 4; 79 mL/d, *P* = .03, *I* ^2^ = 83%), and six interventions that targeted the interpersonal level with/without the individual level (MD = 55 mL/d, CI = 14; 96 mL/d, *P* = .009, *I* ^2^ = 56%). Differences between these four groups were statistically significant (*P* = .004).

#### 3.5.6 Effect by mean age of children

Ten interventions that targeted children with a mean age at baseline of between 2 and 5 years had an overall smaller mean difference between intervention and control group (Table 2 and Figure S5: MD = 16 mL/d, CI = 1; 30 mL/d, *P* = .03, *I* ^2^ = 27%) than had 14 studies that targeted children with a mean age at baseline of between 6 and 12 years (MD = 40 mL/d, CI = 9; 71 mL/d, *P* < .001; *I* ^2^ = 75%). This difference was not statistically significant (*P* = .16).

#### 3.5.7 Sensitivity analyses

The 17 RCTs had overall smaller mean difference between intervention and control groups (Table 2 and Figure S6: MD = 21 mL/d, CI = 6; 36 mL/d, *P* = .005, *I* ^2^ = 42%) than had seven controlled studies with other designs (MD = 57 mL/d, CI = 6; 107 mL/d, *P* = .03; *I* ^2^ = 85%). This difference was not statistically significant (*P* = .19). The overall analysis was repeated with 23 studies, which reported or for which we could calculate mean water consumption in control and intervention groups at follow‐up (Figure S7). Mean difference between control and intervention groups was larger than the overall effect (MD = 37 mL/d, CI = 11; 64 mL/d, *N* = 31 266, *Z* = 2.76, *P* = .006, *I* ^2^ = 86%). Funnel plot inspection indicated that there were fewer small studies that had a negative effect than what would be expected (Figure S8). For two studies,^9^, ^40^ the mean difference between intervention and control groups was larger (outside of the 95% CI) than those of the other studies (Figure S8). When excluding these two studies, the average mean difference between intervention and control groups and heterogeneity of the 22 remaining studies were smaller than the overall effect (MD = 18 mL/d, CI = 5; 31 mL/d, *N* = 15 966, *Z* = 2.81, *P* = .005, *I* ^2^ = 39%).

## 4 DISCUSSION

In this systematic review and meta‐analysis, we investigated the effect of interventions to promote water consumption among children. A total of 47 studies were included that used a large variety of intervention strategies that focused on promoting water consumption, often combined with other diet and/or lifestyle factors. Results from our meta‐analysis indicate that these interventions can lead to a small improvement in water consumption among children. Interventions that focused on diet alone had greater effects on water consumption than had interventions that also included other lifestyle factors. Significant subgroup differences were also found by study setting and socioecological level targeted but not by children's age group, intervention strategy, or study design.

The effect on children's water consumption across the studies included in our review may appear small. However, the size of the effect is also dependent on children's mean water consumption, which varied considerably between the included studies. Our findings confirm evidence from earlier reviews on water consumption in children that found positive but small effects.^24^, ^34^ Vargas‐Garcia et al found an average effect of around 2 oz (60 mL) per day among children older than 3 years.^24^ Cradock et al found effects between 0.5 and 3.5 oz (15–105 mL) per day for children aged 0 to 5 years.^34^ The average overall effect in our review was around 30 mL/d but varied from −28 to 220 mL between studies. Specific interventions may therefore be more effective than others as the lifestyle interventions included in our review showed a wide variation in duration, setting, the lifestyle behaviours focused on, the intervention strategies used to promote water consumption, and the persons or environment targeted by the intervention. Two studies, an RCT by Smit et al that used a peer‐influence intervention strategy and a large non‐RCT by Muckelbauer et al that installed water fountains at schools had a larger effect (220 and 146 mL, respectively) compared with the effect of most other studies included in our review. While these interventions were different in many aspects, the interventions both focused specifically on promoting water consumption and not on decreasing SSB intake or changing other factors.

Many of the interventions included in this review focused on modifying a wide range of lifestyle behaviours among children. These interventions, sometimes called “combined” or “holistic” lifestyle interventions, have been found to be particularly effective in reducing weight among both general populations of children and children with obesity.^23^, ^32^ We, however, found that interventions that specifically focused on diet or beverage consumption on average had a larger effect on water consumption among children than had these combined lifestyle interventions. A reason for this could be that within these broader interventions, the message to drink water receives less attention or gets lost within a multitude of other themes such as physical activity and active play. The design of the intervention strategy itself influences the uptake of messages related to water intake. A combined lifestyle RCT by Contento et al did not have an effect on water consumption but did decrease SSB intake.^48^ This finding illustrates that children may not necessarily replace SSBs by water. Contento et al reported that with regard to the intervention, more time was spent on behaviours related to energy balance and diabetes and that the activities children engaged in were more “memorable” than were behaviours related to water.^48^ When wanting to increase water and decrease SSB consumption, messages that promote water consumption may need to be prioritized alongside messages that limit SSB consumption. Additionally, intervention fidelity might also be lower for combined interventions due to having to divide time and resources over multiple interventions goals.^89^ Intervention goals that are easier to implement might then be prioritized over goals that are more difficult to implement.^61^, ^80^ Siega Riz et al noted that replacing SSBs by water in vending machines was not possible in all schools involved in their combined lifestyle intervention.^80^ De Coen et al found that most schools did not meet their suggested snacks and beverage policy guidelines, which included the installation of water fountains.^61^ Of note is that many countries promote both water and cow's milk as healthy beverages, and these may be competing for messaging space. Especially in settings where malnutrition is a major public health concern, (fortified) cow's milk can contribute important nutrients to a child's diet.^90^, ^91^ Although guidelines mainly recommend including skim or low‐fat milk as part of children's diet,^4^, ^6^ recent observational evidence points towards a negative association between milk fat percentage and children's body mass index (BMI).^92^, ^93^ More research is therefore necessary on the effectiveness of interventions that promote water and/or milk consumption on improving weight and other health‐related outcomes in children.

Although the majority of interventions were based in school settings, interventions in only nonschool settings on average achieved the greatest effect on children's water consumption than did those either in school or in both school and nonschool settings. It might be that there is more room for improvement in water consumption in nonschool settings. Some studies have found that children are more likely to consume SSBs at nonschool settings such as home^94^, ^95^ or recreation venues^96^ and on weekends.^97^, ^98^ Similarly, Vargas‐Garcia et al found that lifestyle interventions in home settings achieved greater reductions in children's SSB consumption than those in school settings.^24^ The greater involvement of parents in home‐based interventions compared with school interventions was suggested to be an important factor in the greater success of these interventions.^24^ Indeed, all nonschool‐based interventions in our meta‐analysis either were based at home or involved parents directly in a community setting. Targeting only the child may not be the best intervention target, as we found that interventions that only targeted the child had a smaller effect than had interventions that also or only targeted parents, peers, and/or the environment. For future interventions, this emphasizes the importance of viewing childhood consumption behaviours within a socioecological framework, as children may be particularly receptive to their social and structural environments. In addition, interventions for young children may be more effective if the caregiver is targeted rather than the child, since the caregiver selects and provides most meals and drinks. So far, research has mainly focused on the association between child‐related factors and water consumption and especially environmental factors have been understudied.^30^ Environmental interventions such as choice architecture interventions may be a promising approach to promote healthy dietary behaviors.^99^, ^100^ Which specific parental and environmental factors need to be targeted in order to improve water consumption among children and which specific components of interventions are most effective in doing so need to be studied in more detail.

### 4.1 Strengths and limitations

To the best of our knowledge, this was the first systematic review and meta‐analysis that focused solely on the effectiveness of interventions in promoting water consumption among children aged 2 to 12 years. The literature search was performed in seven databases, and a rigorous procedure was followed for the inclusion of studies in our review.^101^ However, our review also has some limitations that must be acknowledged. We included RCTs, non‐RCTs, and other quasi‐experimental designs in our systematic review and meta‐analysis. Non‐RCTs and other quasi‐experimental designs are considered to provide lower‐quality evidence and more often show significant results than are RCTs when there are none. We found a lower effect in RCTs compared with other controlled designs in our meta‐analysis, although this difference was not significant. Heterogeneity was moderate to high across the studies included in our review, and subgroup analyses were only partly able to explain this variation. Other differences between studies that were not explored may have explained this variation. Whether or not interventions are theory based may be an important factor in the effectiveness of interventions; however, only two studies included in our meta‐analysis were not theory based. Further, Cochrane advises to have at least 10 studies in each subgroup,^41^ which was not the case for some subgroup analyses in our review. However, findings from subgroup analyses in the meta‐analysis were confirmed in the qualitative subgroup analyses performed with a larger number of studies, which strengthened our findings. Risk of bias was high in most studies, which was, to a large part, due to measurement of outcomes. Retrospective report, which was most commonly used in the studies included in this review, is considered to be imprecise because of poor recall and (parents of) children with low levels of water consumption reporting higher amounts than actual amounts consumed.^102^, ^103^, ^104^, ^105^ For our meta‐analysis, we estimated water consumption in millilitres per day for studies that did not report water consumption in volume per day by using a serving size of 225 mL. This may appear more imprecise compared with volume of water consumed per day, although it is uncertain to what level of precision children and parents can estimate their water consumption.^105^ For younger children aged 2 to 5 years, a serving size of 225 mL may be relatively large; however, because national standardized serving sizes are between 200 and 250 mL, other studies have used similar serving sizes in this age group.^24^, ^49^ Changing serving size to 150 mL for studies with young children that did not report serving size^73^, ^74^ did not change our overall estimate. Finally, we did not include studies published in non‐English languages and studies that were not published in a peer‐reviewed journal; this may have an impact on the generalizability of our results and may have introduced publication bias.^106^

### 4.2 Conclusions

In conclusion, our systematic review and meta‐analysis indicate that interventions can on average lead to a small increase in daily water consumption among children. Dietary interventions and interventions that focus on beverage intake specifically appear to have greater effects on improving children's water consumption than have interventions that focus on both diet and other lifestyle factors. Effects also appeared to vary by study setting and socioecological level targeted. However, more research is needed to further understand the specific intervention elements that have the greatest impact on the water consumption of children. Future research is also needed to determine the effectiveness of these interventions on improving weight and other health‐related outcomes in children.

## CONFLICT OF INTEREST

Dr Franse reports grants from Nestlé Waters while this study was being conducted. Ms Boelens reports grants from Nestlé Waters while this study was being conducted. Dr Fries reports being employed by Societé des Produits Nestlé but has no conflict of interest on the basis of the results of the current review. Dr Constant reports being employed by Societé des Produits Nestlé but has no conflict of interest on the basis of the results of the current review. Dr van Grieken reports grants from Nestlé Waters while this study was being conducted. Dr Raat reports grants from Nestlé Waters while this study was being conducted.

## Supporting information

Table S1: Data used/calculated from studies in meta‐analysis
Table S2: Characteristics and effects of studies included in the review
Table S3: Risk of bias randomized controlled trials
Table S4: Risk of bias non‐randomized controlled trials and non‐controlled quasi‐experimental studies
Figure S1: Effect by focus of intervention
Figure S2: Effect by intervention strategy
Figure S3: Effect by intervention setting
Figure S4: Effect by socio‐ecological level targeted
Figure S5: Effect by mean age children
Figure S6: Effect by study design
Figure S7: Sensitivity analysis random‐effects meta‐analysis of the mean difference in children's water consumption (in ml/day) between intervention and control group (N=24)
Figure S8: Funnel plot of the mean difference (MD) in milliliter water consumption between intervention and control group against the standard error (SE) of the MD of all studies included in the meta‐analysis
