# Supplementary material for: Interventions to increase the consumption of water among children: A systematic review and meta‐analysis
Source: Obes Rev. 2020 Mar 13;21(7):e13015. doi: 10.1111/obr.13015 (PMC7317453; doi:10.1111/obr.13015)
Supplement: Supplementary file 1 — Table S1: Data used/calculated from studies in meta‐analysis Table S2: Characteristics and effects of studies included in the review Table S3: Risk of bias randomized controlled trials Table S4: Risk of bias non‐randomized controlled trials and non‐controlled quasi‐experimental studies Figure S1: Effect by focus of intervention Figure S2: Effect by intervention strategy Figure S3: Effect by intervention setting Figure S4: Effect by socio‐ecological level targeted Figure S5: Effect by mean age children Figure S6: Effect by study design Figure S7: Sensitivity analysis random‐effects meta‐analysis of the mean difference in children's water consumption (in ml/day) between intervention and control group (N=24) Figure S8: Funnel plot of the mean difference (MD) in milliliter water consumption between intervention and control group against the standard error (SE) of the MD of all studies included in the meta‐analysis [file OBR-21-e13015-s001.docx]

| **Databases** | **Before de-duplication** | **After de-duplication** |
| --- | --- | --- |
| embase.com | 10016 | 9854 |
| Medline Ovid | 9281 | 3753 |
| Web of science | 9608 | 3857 |
| PsycINFO Ovid | 1501 | 328 |
| Cochrane CENTRAL | 769 | 210 |
| CINAHL EBSCOhost | 4537 | 1210 |
| Google scholar (top relevant references) | 200 | 134 |
| **Total** | **35912** | **19346** |

**Supplement File 1: Search strategies used**

*New references found: 1673*

**embase.com**

('fluid intake'/exp OR 'beverage'/de OR 'carbonated beverage'/de OR 'carbonated water'/de OR 'drinking water'/de OR 'mineral water'/de OR 'soft drink'/de OR 'sports drink'/de OR 'sweetened beverage'/exp OR 'fruit juice'/exp OR 'fruit and vegetable juice'/de OR tea/de OR 'energy drink'/de OR (((water OR tapwater OR fluid* OR beverage* OR liquid*) NEXT/1 (drinking)) OR ((water OR fluid* OR beverage* OR liquid* OR juice OR drink* OR tea) NEAR/6 (intake* OR consum*)) OR ((carbonat* OR fizzy OR soda OR seltzer OR co2 OR co-2 OR bubble OR sugar* OR nonsugar* OR sweet* OR nonalcohol* OR non-alcohol* OR energy OR soft OR fruit OR sucralose* OR neotame* OR acesulfame* OR saccharin* OR sport*) NEAR/3 (beverage* OR water OR drink*)) OR 'fruit juice*' OR soda OR lemonade OR (bottle* NEAR/3 water)):ab,ti) AND ('child'/de OR 'preschool child'/de OR 'school child'/de or 'primary school'/de OR 'child behavior'/de OR 'child health'/de OR 'child nutrition'/de OR childhood/de OR 'childhood obesity'/de OR (child* OR toddler* OR pre-school* OR preschool* OR schoolchild* OR primary-school* OR elementary-school* OR kindergar*):ab,ti) AND ('risk factor'/exp OR 'social aspects and related phenomena'/exp OR 'social determinants of health'/exp OR 'parent'/exp OR 'lifestyle'/exp OR 'behavior change'/exp OR 'environmental factor'/exp OR 'parental behavior'/exp OR 'child parent relation'/exp OR 'health care policy'/de OR government/de OR 'family life'/de OR advertising/de OR 'television viewing'/de OR 'eating habit'/de OR 'feeding behavior'/de OR 'parental attitude'/exp OR demography/exp OR tax/de OR prevention/exp OR 'health promotion'/exp OR 'health education'/exp OR 'community program'/exp OR 'mass medium'/exp OR 'review'/exp OR 'intervention study'/de OR 'evaluation study'/exp OR 'clinical trial'/exp OR 'caregiver'/de OR (determinant* OR influen* OR factor* OR social OR socio* OR parent* OR mother* OR father* OR maternal* OR paternal* OR lifestyle* OR life-style* OR ((behav* OR habit* OR pattern* OR practic*) NEAR/3 (change* OR diet* OR feeding OR food OR snack* OR intervent* OR health* OR unhealth* OR eating)) OR (environment* NEAR/3 (factor* OR condition* OR home OR school)) OR adverti* OR policy OR policies OR government* OR income OR poverty OR neighbourhood OR neighborhood OR communit* OR sedentar* OR tax OR taxes OR television OR (screen NEAR/3 (view* OR time)) OR Prevent* OR reduc* OR increas* OR promot* OR education OR curriculum OR program* OR polic* OR media OR television OR campaign* OR review* OR intervention* OR initiative* OR strateg* OR evaluation OR trial* OR effect* OR impact* OR ((parent OR role) NEAR/3 model*) OR caregiver* OR ((access OR availab* OR quality OR supply) NEAR/3 (water OR drinkwater))):ab,ti) NOT ([animals]/lim NOT [humans]/lim) NOT ('alcohol consumption'/exp OR ((alcohol OR binge) NEAR/3 (drinking OR consum*)):ab,ti) NOT ([Conference Abstract]/lim OR [Letter]/lim OR [Note]/lim OR [Editorial]/lim) AND [english]/lim

**Medline Ovid**

(Beverages/ OR exp Carbonated Beverages/ OR exp Drinking Water/ OR exp Drinking/ OR exp Mineral Waters/ OR Fruit and Vegetable Juices/ OR Tea/ OR exp Energy Drinks/ OR (((water OR tapwater OR fluid* OR beverage* OR liquid*) ADJ (drinking)) OR ((water OR fluid* OR beverage* OR liquid* OR juice OR drink* OR tea) ADJ6 (intake* OR consum*)) OR ((carbonat* OR fizzy OR soda OR seltzer OR co2 OR co-2 OR bubble OR sugar* OR nonsugar* OR sweet* OR nonalcohol* OR non-alcohol* OR energy OR soft OR fruit OR sucralose* OR neotame* OR acesulfame* OR saccharin* OR sport*) ADJ3 (beverage* OR water OR drink*)) OR fruit juice* OR soda OR lemonade OR (bottle* ADJ3 water)).ab,ti.) AND (exp Child/ OR exp Infant/ OR exp Adolescent/ OR exp "Child Behavior"/ OR exp "Parent Child Relations"/ OR exp "Pediatrics"/ OR "Child Nutrition Sciences"/ OR "Infant nutritional physiological phenomena"/ OR "Child Rearing"/ OR "Child Psychology"/ OR (child* OR toddler* OR pre-school* OR preschool* OR schoolchild* OR primary-school* OR kindergar*).ab,ti.) AND (exp Risk Factors/ OR exp Sociological Factors/ OR exp Social Determinants of Health/ OR exp Parents/ OR exp Life Style/ OR exp Parent-Child Relations/ OR exp Health Policy/ OR exp Government/ OR exp Family Relations/ OR Advertising as Topic/ OR Television/ OR exp Feeding Behavior/ OR exp Demography/ OR Taxes/ OR exp preventive medicine/ OR exp Health Promotion/ OR exp Health Education/ OR Review/ OR Review Literature as Topic/ OR Evaluation Studies/ OR Evaluation Studies as Topic/ OR exp Clinical Trial/ OR Clinical Trials as Topic/ OR Caregivers/ OR (determinant* OR influen* OR factor* OR social OR socio* OR parent* OR mother* OR father* OR maternal* OR paternal* OR lifestyle* OR life-style* OR ((behav* OR habit* OR pattern* OR practic*) ADJ3 (change* OR diet* OR feeding OR food OR snack* OR intervent* OR health* OR unhealth* OR eating)) OR (environment* ADJ3 (factor* OR condition* OR home OR school)) OR adverti* OR policy OR policies OR government* OR income OR poverty OR neighbourhood OR neighborhood OR communit* OR sedentar* OR tax OR taxes OR television OR (screen ADJ3 (view* OR time)) OR Prevent* OR reduc* OR increas* OR promot* OR education OR curriculum OR program* OR polic* OR media OR television OR campaign* OR review* OR intervention* OR initiative* OR strateg* OR evaluation OR trial* OR effect* OR impact* OR ((parent OR role) ADJ3 model*) OR caregiver* OR ((access OR availab* OR quality OR supply) ADJ3 (water OR drinkwater))).ab,ti.) NOT (exp animals/ NOT humans/) NOT (alcohol consumption/ OR ((alcohol OR binge) ADJ3 (drinking OR consum*)).ab,ti.) NOT (letter* OR news OR comment* OR editorial* OR congres* OR abstract* OR book* OR chapter* OR dissertation abstract*).pt. AND english.la.

**PsycINFO Ovid**

(fluid intake/ OR "Beverages (Nonalcoholic)"/ OR exp Water Intake/ OR (((water OR tapwater OR fluid* OR beverage* OR liquid*) ADJ (drinking)) OR ((water OR fluid* OR beverage* OR liquid* OR juice OR drink* OR tea) ADJ6 (intake* OR consum*)) OR ((carbonat* OR fizzy OR soda OR seltzer OR co2 OR co-2 OR bubble OR sugar* OR nonsugar* OR sweet* OR nonalcohol* OR non-alcohol* OR energy OR soft OR fruit OR sucralose* OR neotame* OR acesulfame* OR saccharin* OR sport*) ADJ3 (beverage* OR water OR drink*)) OR fruit juice* OR soda OR lemonade OR (bottle* ADJ3 water)).ab,ti.) AND (100.ag. OR 200.ag. OR "Child Psychology"/ OR (child* OR toddler* OR pre-school* OR preschool* OR schoolchild* OR primary-school* OR kindergar*).ab,ti.) AND (exp Risk Factors/ OR exp Sociocultural Factors/ OR exp Socioeconomic Status/ OR exp Parents/ OR exp LifeStyle/ OR exp Parent Child Relations/ OR exp Health Care Policy/ OR exp Government/ OR exp Family Relations/ OR Advertising/ OR Television/ OR Television viewing/ OR Mass media/ OR exp Food intake/ OR exp Eating behavior/ OR exp Demographic Characteristics/ OR Taxation/ OR exp preventive medicine/ OR prevention/ OR exp Health Promotion/ OR exp Health Education/ OR "literature Review"/ OR Evaluation/ OR Caregivers/ OR (determinant* OR influen* OR factor* OR social OR socio* OR parent* OR mother* OR father* OR maternal* OR paternal* OR lifestyle* OR life-style* OR ((behav* OR habit* OR pattern* OR practic*) ADJ3 (change* OR diet* OR feeding OR food OR snack* OR intervent* OR health* OR unhealth* OR eating)) OR (environment* ADJ3 (factor* OR condition* OR home OR school)) OR adverti* OR policy OR policies OR government* OR income OR poverty OR neighbourhood OR neighborhood OR communit* OR sedentar* OR tax OR taxes OR television OR (screen ADJ3 (view* OR time)) OR Prevent* OR reduc* OR increas* OR promot* OR education OR curriculum OR program* OR polic* OR media OR television OR campaign* OR review* OR intervention* OR initiative* OR strateg* OR evaluation OR trial* OR effect* OR impact* OR ((parent OR role) ADJ3 model*) OR caregiver* OR ((access OR availab* OR quality OR supply) ADJ3 (water OR drinkwater))).ab,ti.) NOT (exp animals/ NOT humans/) NOT (Alcohol Drinking Patterns/ OR ((alcohol OR binge) ADJ3 (drinking OR consum*)).ab,ti.) NOT (letter* OR news OR comment* OR editorial* OR congres* OR abstract* OR book* OR chapter* OR dissertation abstract*).pt. AND english.la.

**CINAHL EBSCOhost**

(MH Beverages OR MH Carbonated Beverages+ OR MH Water OR MH Fruit Juices+ OR MH Tea OR MH Energy Drinks+ OR MH Sports Drinks+ OR (TI ((water OR tapwater OR fluid* OR beverage* OR liquid*) N1 (drinking)) OR ((water OR fluid* OR beverage* OR liquid* OR juice OR drink* OR tea) N5 (intake* OR consum*)) OR ((carbonat* OR fizzy OR soda OR seltzer OR co2 OR co-2 OR bubble OR sugar* OR nonsugar* OR sweet* OR nonalcohol* OR non-alcohol* OR energy OR soft OR fruit OR sucralose* OR neotame* OR acesulfame* OR saccharin* OR sport*) N2 (beverage* OR water OR drink*)) OR fruit juice* OR soda OR lemonade OR (bottle* N2 water)) OR AB (((water OR tapwater OR fluid* OR beverage* OR liquid*) N1 (drinking)) OR ((water OR fluid* OR beverage* OR liquid* OR juice OR drink* OR tea) N5 (intake* OR consum*)) OR ((carbonat* OR fizzy OR soda OR seltzer OR co2 OR co-2 OR bubble OR sugar* OR nonsugar* OR sweet* OR nonalcohol* OR non-alcohol* OR energy OR soft OR fruit OR sucralose* OR neotame* OR acesulfame* OR saccharin* OR sport*) N2 (beverage* OR water OR drink*)) OR fruit juice* OR soda OR lemonade OR (bottle* N2 water))) AND (MH Child+ OR MH Infant+ OR MH Adolescence+ OR MH "Child Behavior+" OR MH "Parent Child Relations+" OR MH "Pediatrics+" OR MH "Child Nutritional Physiology+" OR MH "Child Rearing+" OR MH "Child Psychology" OR TI (child* OR toddler* OR pre-school* OR preschool* OR schoolchild* OR primary-school* OR kindergar*) OR AB (child* OR toddler* OR pre-school* OR preschool* OR schoolchild* OR primary-school* OR kindergar*)) AND (MH Risk Factors+ OR MH Social Determinants of Health+ OR MH Parents+ OR MH Life Style+ OR MH Parent-Child Relations+ OR MH Health Policy+ OR MH Government+ OR MH Family Relations+ OR MH Advertising OR MH Television OR MH Eating Behavior+ OR MH Demography+ OR MH Taxes OR MH Preventive Health Care+ OR MH Health Promotion+ OR MH Health Education+ OR MH Evaluation Research OR MH Caregivers OR TI (determinant* OR influen* OR factor* OR social OR socio* OR parent* OR mother* OR father* OR maternal* OR paternal* OR lifestyle* OR life-style* OR ((behav* OR habit* OR pattern* OR practic*) N2 (change* OR diet* OR feeding OR food OR snack* OR intervent* OR health* OR unhealth* OR eating)) OR (environment* N2 (factor* OR condition* OR home OR school)) OR adverti* OR policy OR policies OR government* OR income OR poverty OR neighbourhood OR neighborhood OR communit* OR sedentar* OR tax OR taxes OR television OR (screen N2 (view* OR time)) OR Prevent* OR reduc* OR increas* OR promot* OR education OR curriculum OR program* OR polic* OR media OR television OR campaign* OR review* OR intervention* OR initiative* OR strateg* OR evaluation OR trial* OR effect* OR impact* OR ((parent OR role) N2 model*) OR caregiver* OR ((access OR availab* OR quality OR supply) N2 (water OR drinkwater))) OR AB (determinant* OR influen* OR factor* OR social OR socio* OR parent* OR mother* OR father* OR maternal* OR paternal* OR lifestyle* OR life-style* OR ((behav* OR habit* OR pattern* OR practic*) N2 (change* OR diet* OR feeding OR food OR snack* OR intervent* OR health* OR unhealth* OR eating)) OR (environment* N2 (factor* OR condition* OR home OR school)) OR adverti* OR policy OR policies OR government* OR income OR poverty OR neighbourhood OR neighborhood OR communit* OR sedentar* OR tax OR taxes OR television OR (screen N2 (view* OR time)) OR Prevent* OR reduc* OR increas* OR promot* OR education OR curriculum OR program* OR polic* OR media OR television OR campaign* OR review* OR intervention* OR initiative* OR strateg* OR evaluation OR trial* OR effect* OR impact* OR ((parent OR role) N2 model*) OR caregiver* OR ((access OR availab* OR quality OR supply) N2 (water OR drinkwater)))) NOT (MH animals+ NOT MH humans) NOT (TI((alcohol OR binge) N2 (drinking OR consum*)) OR AB((alcohol OR binge) N2 (drinking OR consum*))) NOT PT (letter* OR news OR comment* OR editorial* OR congres* OR abstract* OR book* OR chapter* OR dissertation abstract*) AND LA (english)

**Cochrane CENTRAL**

((((water OR tapwater OR fluid* OR beverage* OR liquid*) NEXT/1 (drinking)) OR ((water OR fluid* OR beverage* OR liquid* OR juice OR drink* OR tea) NEAR/6 (intake* OR consum*)) OR ((carbonat* OR fizzy OR soda OR seltzer OR co2 OR "co 2" OR bubble OR sugar* OR nonsugar* OR sweet* OR nonalcohol* OR "non-alcohol*" OR energy OR soft OR fruit OR sucralose* OR neotame* OR acesulfame* OR saccharin* OR sport*) NEAR/3 (beverage* OR water OR drink*)) OR "fruit juice*" OR soda OR lemonade OR (bottle* NEAR/3 water)):ab,ti) AND ((child* OR toddler* OR (pre NEXT/1 school*) OR preschool* OR schoolchild* OR (primary NEXT/1 school*) OR (elementary NEXT/1 school*) OR kindergar*):ab,ti)

**Web of science**

TS=(((((water OR tapwater OR fluid* OR beverage* OR liquid*) NEAR/1 (drinking)) OR ((water OR fluid* OR beverage* OR liquid* OR juice OR drink* OR tea) NEAR/5 (intake* OR consum*)) OR ((carbonat* OR fizzy OR soda OR seltzer OR co2 OR co-2 OR bubble OR sugar* OR nonsugar* OR sweet* OR nonalcohol* OR non-alcohol* OR energy OR soft OR fruit OR sucralose* OR neotame* OR acesulfame* OR saccharin* OR sport*) NEAR/2 (beverage* OR water OR drink*)) OR "fruit juice*" OR soda OR lemonade OR (bottle* NEAR/2 water))) AND ((child* OR toddler* OR pre-school* OR preschool* OR schoolchild* OR primary-school* OR elementary-school* OR kindergar*)) AND ((determinant* OR influen* OR factor* OR social OR socio* OR parent* OR mother* OR father* OR maternal* OR paternal* OR lifestyle* OR life-style* OR ((behav* OR habit* OR pattern* OR practic*) NEAR/2 (change* OR diet* OR feeding OR food OR snack* OR intervent* OR health* OR unhealth* OR eating)) OR (environment* NEAR/2 (factor* OR condition* OR home OR school)) OR adverti* OR policy OR policies OR government* OR income OR poverty OR neighbourhood OR neighborhood OR communit* OR sedentar* OR tax OR taxes OR television OR (screen NEAR/2 (view* OR time)) OR Prevent* OR reduc* OR increas* OR promot* OR education OR curriculum OR program* OR polic* OR media OR television OR campaign* OR review* OR intervention* OR initiative* OR strateg* OR evaluation OR trial* OR effect* OR impact* OR ((parent OR role) NEAR/2 model*) OR caregiver* OR ((access OR availab* OR quality OR supply) NEAR/2 (water OR drinkwater))))) AND DT=(article) AND LA=(english)

**Google scholar**

"water Drinking|consumption|intake" child|children determinants|determinant|influence|factors

| **Table S1: Data used/calculated from studies in meta-analysis** | | |
| --- | --- | --- |
| **Author** | **Choices made (if applicable)** | **Calculations (if applicable)** |
|  |  |  |
| Baranowski | Last time point | 1 ounce=29.57 ml |
| Contento |  | Meals and in between meals added together, for SD1+2=√SD1²+SD2². Multiplied by days and ml; 8 ounce glass=236.59 ml (in paper) |
| Crespo | Last time point  Fam+comm intervention | Glass=225 ml (self-estimated) |
| De Bourdeaudhuij | All countries together | Divided by 7 days and * 225 ml (self-estimated). SE recalculated to SD. Estimated same number of boys and girls in intervention and control group (unreported in paper) |
| de Silva-Sanigorski | | Glass/cup is 250 ml in paper |
| Elder |  | 8-oz glass of water=236.59 ml (in paper) |
| Franken |  | Glass=225 ml (self-estimated). N, Mean and SD at follow-up sent by author |
| Franks | Last time point (12 months post baseline), info+w+social group | SE=taken as SD, because very wide and in other paper Lahlou, 2015 reported as SD |
| James |  | 250 ml glass (in paper) |
| Klesges | Last time point (2 year follow-up); | Serving=225 ml (self-estimated) |
| Majumdar |  | Conservatively 5 ounce per point (4=20 ounce); 1.75 day per point (4=7 days). 1 ounce=29.57 ml |
| McGarvey |  | 0.75 serving per point because mean is around 4 points (3 glasses) (1=0 times and 6=5 or more times). 1 occasion=225 ml (self-estimated) |
| McGowan |  | 1 occasion=225 ml (self-estimated) |
| Muckelbauer | | Glass=200 ml (in paper) |
| Novotny |  | Cups (in paper)=236.59 |
| Pinket |  | SD and N sent by author |
| Siega-Riz |  | Grams=ml |
| Smit |  | Glass=225 ml (self-estimated) |
| Story |  | 1 time=225 ml (self-estimated) |
| Taylor |  | Divided by 3 days and * 225 ml (self-estimated) |
| van de Gaar | Child report |  |
| Veitch | Last time point (20 months after start) | |
| Vereecken | Parent report (teacher report is negligible = 0.08 ml) |  |

| Table S2: Characteristics and effects of studies included in the review | | | | | | | | | |
| --- | --- | --- | --- | --- | --- | --- | --- | --- | --- |
| Author, year & country | **Design & name** | **Description of the intervention (I) and control (C) condition** | **Length of intervention and follow-up time** | **Age in years** | **Specific characteristics of population** | **Number participants (& clusters)** | **Water Assessment & outcome** | **Significant effect** | **Participation & retention rate** |
| Abi Haidar, 2011  Lebanon | Pretest-posttest  Jarrib Baleha ‘try without it’ | I= Education sessions at school for children offered by a graduate student.  Theory used=Health Belief Model  Focus=On beverage consumption  Freq=Four 50-min sessions  C=NA | L=2 weeks  FU=2 weeks | M=10.4 | Public school children (lower SES) | I=93  C=NA | FFQ  Cups/day | Yes | P=92%  R=85% |
| Baranowski,  2011  USA | RCT  Diab & nano | I= Two educational computer games, Diab or Nano, for children offered at home and played in sequence.  Theory used=Social cognitive, self-determination, and persuasion theories  Focus=On diet and lifestyle  Freq=9 sessions of 40 min game-play  C=Knowledge-based nutrition computer game offered at school and 8 sessions of game-based websites (each related to diet, PA and obesity) | L=as long as desired  FU=two months (2 measure points) | R=10-12 | 50-95th percentile BMI, >50% ethnic minority communities, access high-speed internet | I=93  C=40 | 3 24-hr dietary recalls  Ounces/day | No | P=51%  R=93% |
| Bea, 2015  USA | Pretest-posttest  SNAP-Ed | I=Education teachers (TTT) administrators, staff and children at school offered by a professional nutrition educator.  Theory used=no theory reported  Focus=On diet  Freq=1-2 hr initial training, new material provided monthly  C=NA | L=varied between classrooms FU=6 months | M=9.7 | Children who qualified for the Supplemental Nutrition Assistance Program-Education Program (SNAP Ed) | I=770  C=NA | 1 24-hr dietary recall  Times consuming water yesterday | No | P=72%  R=90% |
| Beets, 2014  USA | Pretest-posttest  healthy lunchbox challenge | I=Incentive program for children, their parents and the staff at summer day camps with a competition to reach group-based healthy eating goals.  Theory used=Behavioral choice theory and goal-setting theory  Focus=On diet  Freq=4 days/week and 8 hr/day  C=NA | L=8 weeks  FU=3 years (3 measure points) | M=7.8  R=4-12 | 46% white non-Hispanic, 6-15% in poverty | I=~550  C=NA | 16-day observation  Proportion children reaching target/day | No | P=no info  R=no info |
| Beets, 2017  USA | Cluster RCT  STEPs-HE | I=Restructuring the afterschool environment by incorporating new routine practices, trainings and boosters sessions offered by research staff for site-leaders and staff, availability of healthy foods and development of guidelines for healthy menu and PA.  Theory used=Maslow’s hierarchy of needs, nonspecific hypothesis in psychotherapy.  Focus=On diet  Freq=3 hr initial training, 4-6 booster sessions 20-30 min.  C=Delayed group, same intervention | L=2 years  FU=2 years | M=8 | Around a third African American | I=894 (10 afterschool programs)  C=760 (10 afterschool programs) | 4-day observation  Observing cups of water served during snack time/day (Y/N) | Yes | P=100%  R=94% |
| Bender, 2013  USA | Pretest-posttest  Vida Saludable | I=Education as interactive group lessons (phase 2) at a health center and group community activities for mothers and children offered by trained a promotora ((community health advisor) tailored to Hispanic families.  Theory used: Social cognitive theory  Focus=On diet and lifestyle.  Freq=Phase 1: four times biweekly and phase 2: six times monthly  C=NA | L=9 months FU=15 months | M=3.6 | low-income Mexican mothers with children included | I=33  C=NA | 1 24-hr dietary recall  Ounces/day | Yes | P=no info  R=70% |
| Brand, 2017  Germany | Pretest-Posttest | I=Multifaceted intervention to restructure the kindergarten environment by improvement of nutritional standards for kitchen staff, education, availability of healthy foods and water, policy changes, trying dishes with children, preparing mails, information for parents and availability of tap water (group A) or by a nutrition training for kitchen staff (group B).  Theory used=no theory reported  Focus=On diet  Freq= Group A: no info, group B 25 h training group C, no info  C=NA | L=5 years  FU=12 months | R=3-6 | 1 parent included for each child; more than 50% migrant background. | I=Group A: 171 group B: 80 group C: 123  C=NA | FFQ  Times/week | Yes | P=27-47% R=75-86% |
| Canavera, 2009  USA | Pretest-posttest | I=Multifaceted intervention using education sessions for children at school by physical or health education teachers.  Theory used=Social cognitive theory  Focus=On diet and lifestyle.  Freq=4 modules in 12 sessions  C=NA | L=12 weeks  FU=12 weeks | R=10-12 | Mainly white children | I=122  C=NA | 1 24-hr dietary recall  Glasses/day | Yes | P=no info  R=no info |
| Contento, 2010  USA | Cluster RCT  Choice, Control, and Change | I=Education at school for children by science teachers about the interactions between biology, personal behavior and the environment and personal agency regarding healthy nutrition and activity, TTT for teachers and research staff helped to prepare lessons/give feedback.  Theory used=Social cognitive theory, self-determination theory, Questioning, Experimenting, Searching, Theorizing, and Applying to Life (QuESTA), guided goal-setting  Focus=On diet and lifestyle  Freq= 24 lessons of 45 min  C=Standard science curriculum of equal intensity and duration, receiving C3 the next term as a delayed intervention | L=8-18 weeks  FU=8-10 weeks | M=12 | Schools in underserved low-income neighborhoods | I=460 (5 schools)  C=437 (5 schools) | FFQ  8-ounce glasses per day & number days/week with meals and in between meals | No | P=99%  R=77% |
| Crespo, 2012  USA | Cluster RCT  Aventuras para Niños | I= Fam-only: Multifaceted family education intervention consisting of home-visits by trained promotoras and increasing active play and decreasing SSBs and television viewing.  Comm-only: Aimed to restructure the school/community environment for children and parents by availability of water, policy changes, healthy eating at school, and social marketing strategies.  Fam+comm: Both fam-only and comm-only.  Theory used: ecological systems theory, health belief model, social cognitive theory, structural model of health behavior.  Focus=On diet and lifestyle.  Freq=Promotoras received 22 hours of training in 11 sessions; 1 home-visit per month was to be scheduled.  C=No intervention | L=7 months (fam-intervention); 3 years (com-intervention)  FU=3 years (4 measure points) | M=5.9 | Largely Hispanic | I=Fam-only=96 (3 schools) Fam+Com=83 (3 schools)  Com-only=128 (schools)  C=134 (4 schools) | FFQ  Glasses/day | No | P=97%  R=55% |
| de Bock, 2016  Germany | Cluster RCT | I=Activities and meal preparation at school by trained nutrition expert for children and parents.  Theory used=Social learning theory and exposure effect theory  Focus=On diet  Freq=15 2 hr sessions  C= Delayed, received the same intervention 6 months later than the intervention arm | L=6 months  F=12 months | M=4.3 | Third from immigration background | I=194 (10 schools)  C=183 (8 schools) | FFQ  Glasses/day | No | P=87%  R=81% |
| De Bourdeaudhuij,  2015  Belgium, Cyprus, Estonia, Germany, Hungary, Italy, Sweden & Spain | nRCT  IDEFICS | I=Multifaceted approach to restructure the school and community environment for children by availability of water, school food policy changes, social marketing changes, creation of community platforms with local public authorities and others, environmental changes in the community and education and activities offered by researchers, teachers and parents who received training.  Theory used=Socio-ecological model, intervention mapping  Focus=On diet and lifestyle.  Freq=10 modules  C=No intervention. | L=2 years  FU= 2 years | M=6 | General population | I=5727 (8 region counties)  C=5314 (8 region counties) | FFQ  Frequency/week | No | P=53%  R=68% |
| de Coen, 2012  Belgium | Cluster RCT POP Project | I=Multifaceted intervention consisting of a website for parents, education and social marketing campaign for children at schools and community by teachers/other school staff who received training material and key community stakeholders who applied the social marketing campaign for children.  Theory used=Socio-ecological model, concept mapping, healthy school framework  Focus=On diet and lifestyle  Freq=4 meetings with teachers, 5 healthy weeks with weekly 1 hr lesson. Contact regional health boards at least twice/year  C=No intervention | L=2 years  FU=2 years  2 years (2 measure points) | M=5 | Low, middle and high SES communities | I=396 (3 communities)  C=298 (3 communities) | FFQ  Ml/day | No | P=63%  R=44% |
| De Silva-Sanigorski, 2010  Australia | Repeated cross-sectional  controlled  Romp & chomp | I=Multifaceted community-based intervention focused on social (parents) and structural environments of children consisting of a social marketing campaign and restructuring the preschool environment by availability of water and policy changes.  Theory used=Socio-ecological model  Focus=On diet and lifestyle  Freq=Various length and duration  C=Comparison communities drawn from local government areas | L=4 years  FU=control group only measured at follow-up and intervention 3 years (2 measure points) | 2 and 3.5 | General population | I=344  C=696 | 1 24-hr recall  Servings previous day | No | P=no info  R=NA |
| Elder, 2014a  USA | Cluster RCT  MOVE/me Muevo | I= Multifaceted family education intervention at recreation centers consisting of workshops, a home-visit and phone consultation by trained health coach and restructuring environment of the recreation center in PA and healthy foods and beverages offered.  Theory used=Socio-ecological model  Focus=on diet and lifestyle.  Freq=4 1.5 h workshops, 1 h home visit, 2 10 min phone calls  C=take-home information and giveaways on non-obesity related topics | L =2 years  FU=2 years (3 measure points) | M=6.6 | Largely Hispanic | I=238 (15 recreation centers)  C=256  (15 recreation centers) | FFQ  servings/day | No | P=47%  R=91% |
| Elder, 2014b  USA, Mexico | nRCT  Agua para ninos (Water for Kids) | I= Multifaceted intervention consisting of positive reinforcement for water consumption at schools through teachers who were trained appraisals coupled with reduced access to restructuring the environment to reduce barriers to obtain water by distribution water and water bottles, pee meters installed, class activities and education for children at school and information for parents.  Theory used=Operant conditioning  Focus= On beverage consumption  Freq=1 hr teacher training, weekly lessons about water consumption (20-30 min)  C=No info | L=8-12 week  FU=6 or 10 weeks | Elementary school  age | Mexican-American or Mexican background | I=2 schools  C=2 schools (no data individual children) | 10-day observation  Having water bottle on desk, water consumed at lunch | Yes | P=NA  R=NA |
| Franken, 2018  Aruba | Cluster RCT  Share H2O | I=Identifying and training the most influential children as PIs to promote water consumption among children and asking the PIs to promote water consumption among children in their social networks at schools and handing out reusable water bottles.  Theory used=Social network principles  Focus=On beverage consumption  Freq=90 min training, received reusable water bottle, follow-up training in weeks two and five  C=No intervention | L= 8 weeks  FU=8 weeks | M=11.4 | General population | I=192 (2 schools)  C=185 (2 schools) | FFQ  Glasses/day | No | P=83%  R=96% |
| Franks, 2017  Poland | nRCT | I=group 1: Child and carer attended online coaching sessions from home; group 2: Child and carer attended online coaching sessions and restructuring the home environment by receiving water and half of both groups had access to online support. Theory used=Installation theory  Focus=On beverage consumption.  Freq=2 sessions per week  C=No intervention | L=3 weeks  FU=12 months (6 measure points) | M=4.4 | Children who drank low quantities of water and high quantities of SSBs | I1a=64 I1b=72 I2a=65  I2b=72  C=61 | 7-day dietary record  Ml/day | Yes | P=94%  R=76% |
| Freedman, 2010  USA | Pretest-posttest  Snack Smart | I=Workshops taught by trained nutrition students for children and parents in public libraries.  Theory used=Social cognitive theory  Focus=On diet  Freq=5 workshops 6 hr total  C=NA | L=3 weeks  FU=3-4 months (3 measure points) | M=11.1 | Ethnically diverse | I=14  C=NA | FFQ  Frequency/day | No | P=25%  R=29% |
| Galvan, 2016  Mexico | Pretest-posttest | I=Social marketing promotion campaign at school to raise awareness on healthy eating and water drinking and make a daily plan of fruit and vegetables to bring to school and monitoring by teachers.  Theory used: Social marketing  Focus=On diet  Freq=30 min session  C=NA | L=3 months  FU=3 months (2 measure points) | R=6-12 | 56% private schools, rest public schools | I=226  C=NA | 1-day observation  Ml/day | Yes | P=no info  R=92% |
| Giles, 2012  USA | Delayed cluster- RCT  Out of School Nutrition and Physical Activity Initiative | I=Restructuring the environment by implementing menu changes and water-delivery systems at afterschool programs for children and training about goal setting, problem solving and implementing policies and communication strategies related to PA and nutrition for program directors and staff given by the research team.  Theory used=Social-ecological model and community-based participatory approach  Focus=0n beverage consumption.  Freq= three sessions 3 h  C=Delayed group, same intervention | L=6 months  FU=6 months | M=7.8 | Ethnically and economically diverse populations | I=10 programs  C=10 programs (no data individual children) | 5-day observation  Ounces served/day | Yes | P=65%  R=80% |
| Gittelson, 2010  Hawaii | nRCT  Healthy Foods Hawaii | I=Restructuring the environment by increasing availability of healthy foods in stores and social marketing strategies including in-store posters, educational displays, stocking of healthy foods and shelf labels, cooking demonstrations and taste tests all aimed at parents.  Theory used=no theory reported  Focus=On diet  Freq=4 phases with 4-6 cooking demonstrations  C=No intervention | L=9-11 months FU=9-11 months (2 measure points) | M=9.9 | Mother-child dyads from low-income multiethnic communities | I=64  C=53 | FFQ  Times/week | Yes | P=80%  R=no info |
| Hornsby, 2017  USA | Pretest-posttest  Cavities Get Around | I=Multifaceted social marketing campaign aimed at children and parents focused at oral health and replacing SSBs by water which included advertising, social media, educational programs, text messaging, raise awareness, community engagement and education through promotores de salud (community health worker).  Theory used=Stages of change from the trans-theoretical Model  Focus=On beverage consumption  Freq=Various length and duration  C=NA | L=Various length and duration  FU=1.5 years (2 measure points) | 0.5-6 | Low-income parents selected who had child with appropriate age | I=600  C=NA | FFQ  Daily consumption (Y/N) | Yes | P=no info  R=no info |
| James, 2004  UK | Cluster RCT  CHOPPS | I=Education and activities for children in classroom about SSBs and water consumption delivered by researchers and assisted by teachers.  Theory used=no theory reported  Focus=On beverage consumption  Freq=4 sessions  C=No intervention | L=1 school year  FU=1 year (2 measure points) | M=8.7 | General population | I=295 (15 school classes)  C=279 (14 school classes) | 3-day dietary record  Glasses/3 days | No | P=71%  R=55% |
| Kaufman Shriqui, 2016,  Israel | Cluster RCT | I=Education to mothers and children by trained dieticians, economists and PA teachers on healthy, affordable nutrition and PA teachers were trained to compliment children who had healthy lunch.  Theory used=social-ecological model  Focus=On diet and lifestyle.  Freq=10 weeks 45 min lectures/week, weekly newsletter, 3 workshops  c=only PA lessons | L=3 months  FU=  6 months (3 measure points) | M=5.3 | Low SES | I=66 (4 schools)  C=165 (7 schools) | FFQ  Habitual water drinking (Y/N) | Yes | P=63%  R=97% |
| Klesges, 2010  USA | RCT  Memphis GEMS | I=Group behavioral counselling for children and parents by community center staff for girls and carers at community centers and educational field-trip.  Theory used=Social cognitive theory  Focus=On diet and lifestyle.  Freq=90 minute sessions weekly for 14 weeks and then monthly for 20 months  C=3-monthly sessions focused on self-esteem and social-efficacy | L=2 years  FU=2 years (3 measure points) | M=9.3 | African-American girls with BMI at or higher than 25th percentile for age | I=116  C=127 | 3 24-hr recall  servings/day | Yes | P=65%  R=80% |
| Laurence, 2007  Australia | Repeated cross-sectional  Fresh Kids | I=Multifaceted school intervention for children and staff coordinated by a community dietician based on social marketing and restructuring the environment which is incorporated in a municipal plan, nomination of lead teacher within each school, fruit and vegetable wholesalers and handing out seasonal free fruit, newsletters to parents, scheduling fruit breaks, development of school fruit/water policies nutrition education curriculum provided to schools, and water bottles printed with student design logos.  Theory used=WHO Health Promoting Schools Framework  Focus=On diet  Freq= Children receive seasonal free fruit 2-4 times a year  C=NA | L=4 years  FU=3 years (4 measure points) | primary-school students | Schools are located within an urban multicultural municipality in which a high proportion of residents experience socioeconomic disadvantage | I=4 schools  C=NA  (no data individual children) | Lunchbox audit by teachers  Water in bottle on desk/in lunchbox | Yes | P=90%  R=81% (but in only 2/4 schools) |
| Majumdar, 2013  USA | nRCT  Creature 101 game | I=Educational computer game for children at school on benefits of drinking water and healthy diet/nutrition/PA, analyzing own eating and PA behaviors and set personal goals, reporting on own goal progress and lessons.  Theory used=Social cognitive and self-determination theories  Focus=On diet and lifestyle  Freq=9 sessions of 30 min  C=Month "Whyville" computer game about broad range of topics (excluding nutrition games) | L=1 month  FU=1 month (2 measure points) | M=11.3 | Public schools in low-income areas NYC | I=182 (4 schools)  C=159 (2 schools) | FFQ  Water consumption frequency | No | P=90%  R=67% |
| McGarvey, 2004  USA | nRCT  Fit WIC | I=Education groups and individual nutrition session with trained nutritionists for parents at WIC center; staff participated also in nutrition/PA training and were asked to model behaviors to parents.  Theory used=Social cognitive theory and self-efficacy theory  Focus=On diet and lifestyle  Freq=Educational groups every 2 months and individual session every 6 months  C=Standard WIC interventions (nutrition education every 2 months and individual session every 6 months), but lacked interventions content and supportive staff. | L=1 year  FU=1 year | M=3.1 | Ethnically diverse, low income. | I=121 (1 center)  C=65 (1 center) | 1 24-hr dietary recall  Times/day | Yes | P=85%  R=65% |
| McGowan, 2013  UK | Cluster RCT  Healthy feeding habits | I=Education sessions by trained researchers to parents at home on offering fruit and vegetables, healthy snacks, and healthy drinks in which they received information about a.o. habit formation and healthy feeding.  Theory used=Habit model  Focus=On diet  Freq=4 visits of 1 h  C=No intervention | L= 8 weeks  FU=8 weeks (2 measure points) | M=3.2 | General population | I=51 (3 children’s centers)  C=55 (3 children’s centers) | FFQ  Occasions/day | Yes | P=60%  R=84% |
| Muckelbauer, 2009 Germany | nRCT | I=Multifaceted intervention for children and teachers consisting of restructuring the environment by installation water fountain(s) in schools, each child received a plastic water bottle and teachers were encouraged to organize filling of the water bottles each morning and teachers gave lessons to children on water needs.  Theory used=Theory of planned behavior, goal-setting strategy  Focus=On beverage consumption  Freq=4 lessons 45 min, booster session after 3 months, water bottle at beginning and after 5 months  C=No intervention | L= 10 months FU=10 months (2 measure points) | M=8.3 | Children from deprived neighborhoods | I=1070 (17 schools)  C=917 (15 schools) | 1 24-hr dietary recall  Glasses/day | Yes | P=60%  R=84% |
| Novotny, 2018  USA Islands | Cluster RCT  Children’s Healthy Living Program | I=Multifaceted intervention at preschool with 4 strategies: organizational policy change (preschool wellness policies), restructuring the environment (increasing access to healthy foods and environment for safe play), social marketing, and training (role models, parents, and teachers).  Theory=Social-ecological model, ANGELO framework  Focus=On diet and lifestyle.  Freq= Various length and duration  C=Delayed optimized intervention: activities that worked best were implemented after time period study | L= 2 years  FU=2 years (2 measure points) | M=5.4 | 64% indigenous children | I=1342 (9 communities)  C=1295 (9 communities) | 2-day dietary record  Cups/day | No | P=94%  R=71% |
| Patel, 2011  USA | nRCT | I=Multifaceted intervention at school consisting of restructuring the environment provision of tap water in school cafeteria, distribution of reusable water bottles to all school staff and students, implementation of school-wide social marketing promotional activities, education regarding the benefits of drinking water (posters, flyers, 1 education session to 30 parents).  Theory used=no theory reported  Focus=On beverage consumption  Freq=1 education session parents/staff/children  C=No intervention | L=5 weeks  FU=2 months (3 measure points) | M=12.8 | Low income, ethnically diverse | I=377 (1 school)  C=440 (1 school) | 1 24hr recall  Drank water at school (Y/N) | Yes | P=78%  R=90% |
| Patel, 2016  USA | Cluster RCT | I=Multifaceted intervention based on restructuring the environment by installation (filtered) water dispensers or coolers, cups provided, social marketing promotional activities (posters, audio announcements, newsletters, prices) at school and provision of rewards at school.  Theory=Trans theoretical model, P’s of social marketing, conceptual framework by Brennan et al. prevention childhood obesity.  Focus=On beverage consumption  Freq= No info  C=Only traditional water fountains | L= 6 weeks  FU=6 weeks (2 measure points) | M=12.7 | Low income, ethnically diverse | I=dispenser=203 (4 schools) cooler=193 (4 schools)  C=199 (4 schools) | FFQ  Lunchtime water consumed (Y/N) | Yes | P=84%  R=98% |
| Pinket, 2016  Belgium, Bulgaria, Germany, Greece, Poland, Spain | Cluster RCT  Toybox | I=Multifaceted intervention at for children and staff at kindergarten consisting of trained teachers with material to provide education, classroom activities, restructuring the environment by installation of water stations, newsletters for parents and material to take home.  Theory used=PRECEDE-PROCEDE model, Intervention Mapping  Focus=On diet and lifestyle  Freq=2 teacher training sessions, 11 games for children  C=Normal routine | L=24 weeks; drinking module in week 1 and 4 and 17 and 18  FU=1 year | M=4.7 | Five target municipalities were chosen per SES status in each country | I=3080 (6 countries)  C=1647 (6 countries) | FFQ  Ml/day | No | P=63%  R=59% |
| Rangelov, 2018  Switzerland | RCT  FAN Social Marketing program | I=Web-based social marketing intervention for children and parents using a website and forum (web-only) with SMS (intervention group 1) or with e-mail (intervention group 2) as reminders with tailored communication about nutrition and PA.  Theory used=Social Marketing benchmarks.  Focus=On diet and lifestyle  Freq=Different weekly themes, weekly reminders via SMS/email  C=NA | L= 8 weeks  FU=8 weeks | M=8.5 | General population | I=web+SMS=  194 Web+email=196  Web-only=218 C=NA | 7-day dietary record  Times/day | No | P=5%  R=83% |
| Rauba, 2017  USA | Pretest-posttest  Energy Up | I=A lesson created by school Wellness Committee (parents, teachers, health professionals) filling plastic bags with sugar and pieces of candy found in SSBs and comparing them to daily limit for age group, restructuring the environment by installation of drinking fountains, distribution of water bottles, and social marketing by school announcements, posters placed in school.  Theory used=no theory reported  Focus=On beverage consumption  Freq=1 lesson, weekly school announcements  C=NA | L=4 months  FU=6 months | 3^rd^ - 5^th^ grade students | Children of a suburban school | I=211  C=NA | 1 24-hr dietary recall  Times/day | No | P=no info  R=99% |
| Romo, 2018  Equador | Pretest-posttest | I=Intervention for children by teachers trained to deliver an educational curriculum, activities, games, traffic light stickers to indicate behavior change all focused on drinking water instead of SSBs engaging in PA and less screen time. Only in the enhanced intervention group parents were trained by teachers and received a workbook with activities for to do with their children at home and refrigerator magnets as rewards for completed activities.  Theory used=Social cognitive theory  Focus=On diet and lifestyle  Freq=1 hr per day on activities  C=NA | L= pilot 3 months and enhanced intervention 7 months  FU=pilot 3 months and enhanced intervention 7 months | M=3.5 | Children from municipal preschools | I=pilot group=132 enhanced group=144  C=NA  (9 schools in total) | FFQ  Daily consumption (Y/N) | Yes | P=95%  R=90% |
| Siega-Riz, 2011  USA | Cluster RCT  HEALTHY study | I=Restructuring the environment by changes in the quantity and quality of food offered at school, including cafeteria and afterschool snacks, a la carte and vending machines and messaging, cafeteria-based educational events, taste tests, food staff training sessions, nutrition education classroom and reinforcing healthy behavior through messages, images, events and activities.  Theory used: Social marketing principles  Focus=On diet and lifestyle  Freq=No info  C=NA | L= 2.5 years  FU=2.5 years (5 school semesters) | M=11.3 | Low income, ethnically diverse | I=1964 (21 public middle schools)  C=1944 (21 public middle schools) | FFQ  Grams/day | Yes | P=no info  R=85% |
| Smit, 2016  The Netherlands | Cluster RCT  Share H2O | I=Intervention for children by using PIs chosen based on nominations of class-mates were trained to emphasize benefits of water, consume more water themselves, promote water consumption in social network at school.  Theory used=Self-persuasion theory, self-determination theory  Focus=On beverage consumption  Freq= 1 session of 90 min, 2 follow-up sessions  C=No intervention | L=9 weeks  FU=8 weeks (2 measure points) | M=10.8 | Urban and suburban primary schools | I=106 (2 primary schools)  C=104 (2 primary schools) | FFQ  Glasses/day | Yes | P=14%  R=86% |
| Story, 2012  USA | Cluster RCT  Bright Start | I=Restructuring the school environment including parent involvement and teachers trained to deliver PA in class, food service staff and teachers trained to offer healthy foods and eliminate unhealthy foods, students encouraged to drink water, family events with information, activities, goal setting, take-home incentives and follow-up telephone calls parents.  Theory used=Formative assessment, Social Cognitive Theory  Focus=On diet and lifestyle  Freq=3 family night events, 1 summer event  C=No intervention | L=14+31 weeks  FU=1.5 years (2 measure points) | M=5.8 | All American-Indian Children | I=267 (8 schools)  C=187 (6 schools) | FFQ  Times drinking bottled water/day | No | P=96%  R=92% |
| Taylor, 2007  New Zealand | nRCT  Apple project | I=Multifaceted intervention for children at school consisting of restructuring the environment by increased availability of PA, cooled water filters, science lessons with lessons and activities on healthy eating and PA implemented by community activity coordinators.  Theory used=no theory reported  Focus=On diet and lifestyle  Freq=No info  C=No intervention | L=2 years  FU=2 years (3 measure points) | M=7.7 | Predominantly white | I=151 (4 schools)  C=137 (3 schools) | 3-day FFQ  Servings/3 days | No | P=90%  R=62% |
| Van de Gaar, 2014  The Netherlands | Cluster RCT  the water campaign | I=Multifaceted intervention for children and parents consisting of lessons at school combined with community social marketing campaign and activities that promote water consumption (various e.g. provision water bottle, pimp water bottle, water theme week, posters).  Theory used=Intervention mapping, social marketing  Focus=On beverage consumption  Freq=Various length and duration  C=Regular health promotion program | L=1 year  FU=1 year (2 measure points) | R=6-12 | Low income, ethnically diverse | I=182 (2 schools)  C=205 (2 schools) | FFQ  L/day | No | P=55% (parents, 84% (children)  R=62% (parents), 75% (children) |
| Veitch, 2011  The Netherlands | Cluster RCT  Doit intervene-tion | I=Lessons for children at school by biology and physical education teachers to raise awareness of energy balance behaviors and to facilitate behavioral changes and restructuring the environment by advice to school canteens in availability of foods/drinks.  Theory used=Intervention mapping  Focus=On diet and lifestyle  Freq=11 lessons  C=No intervention | L=8 months  FU=20 months (4 measure points) | M=12.7 | General population | I=402 (10 schools)  C=354 (8 schools) | FFQ  Ml/day | No | P=74%  R=79%-93% |
| Vereecken, 2009  Belgium | Cluster RCT  Beastly Healthy at School | I=Education and activities at preschool for children by trained school staff, newsletters and evenings for parents, training principals and schools staff a.o about role modeling.  Theory used=Intervention mapping protocol, experiential education and developmental education  Focus=On diet  Freq=2 day training school staff  C=No intervention | L=no info  FU=6 months (2 measure points) | ≥2.5 | General population | I=308 (8 preschools)  C=168 (8 preschools) | FFQ  Ml/day | No | P=62%  R=86% |
| Waters, 2017  Australia | Cluster RCT  fun ‘n healthy in Moreland | I=Multifaceted content program determined by school strategies about increasing fruit and vegetables, water, PA and self-esteem among children and support by community development workers.  Theory used=WHO Health Promoting Schools Framework  Focus=On diet and lifestyle  Freq=No info  C=No intervention | L=3.5 years FU=3.5 years  (3 measure points) | M=5-12 | General population | I=1426 (12 schools)  C=1539 (10 schools) | 1-day 24hr dietary record  Water in lunchbox/canteen order | Yes | P=45%  R=95% |
| Wolfe, 2018  USA | Pretest-posttest  Choose Health: Food, Fun, and Fitness | I=Nutrition education consisting of interactive nutrition activities, preparing or tasting healthy snacks, playing active games, setting weekly goals, take home materials taught by community educators at schools and afterschool programs for children and parents.  Theory used=Social cognitive theory, experiential learning model  Focus=On diet and lifestyle  Freq=6 weekly lessons of 45-90 min  C=NA | L= 6 weeks  FU=6 weeks  (2 measure points) | 3rd - 5th grade students | Low income ethnically diverse | I=686  C=NA | FFQ  Frequency drinking water | Yes | P=No info  R=No info |
| *+=non-significant positive, ++ significant positive, -non-significant negative, / unclear non-significant; a.o=amongst others; BMI=body mass index; C=control; comm=community; fam= family; FFQ=food frequency questionnaire; FU=follow-up; h=hours; I=intervention; L=length/liters; min=minutes; ml=milliliter; NA=not applicable; nRCT=non-randomized controlled trial; NYC=New York City; P=participation rate; PA=physical activity; PI=peer influencer; R=range; RCT= randomized controlled trial; R=retention rate; SES=socioeconomic status; SSB=sugar sweetened beverage; TTT=teach the teacher; USA Islands=Alaska, American Samoa, Commonwealth of the Northern Mariana Islands, Guam, and Hawaii; WIC=nutritional program for women infants and children. | | | | | | | | | |

| Table S3: Risk of bias randomized controlled trials | | | | | | | |
| --- | --- | --- | --- | --- | --- | --- | --- |
|  | **Random sequence generation bias** | **allocation concealment bias** | **Blinding of participants, personnel and outcome assessors bias** | **Incomplete outcome bias** | **Selective outcome reporting bias** | **Other sources of bias (measurement outcomes, departures intended interventions, choice statistical methods)** | **Overall risk of bias** |
| Baranowski,2011 | unclear | unclear | low | low | low | low | **unclear** |
| Beets, 2017 | low | low | low | low | low | low | **low** |
| Contento, 2010 | unclear | low | unclear | low | low | high | **high** |
| Crespo, 2012 | unclear | unclear | unclear | high | low | high | **high** |
| de Bock, 2016 | unclear | low | unclear | high | high | high | **high** |
| de Coen, 2012 | high | low | high | high | high | high | **high** |
| Elder, 2014 | unclear | unclear | unclear | low | high | high | **high** |
| Franken, 2018 | unclear | low | low | low | low | high | **high** |
| Giles, 2012 | unclear | low | unclear | low | low | low | **unclear** |
| James, 2004 | low | low | low | high | low | unclear | **high** |
| Kaufman Shriqui, 2016 | low | low | low | low | low | high | **high** |
| Klesges, 2010 | low | unclear | low | low | low | low | **unclear** |
| McGowan, 2013 | low | low | high | low | low | high | **high** |
| Novotny, 2018 | low | unclear | unclear | low | low | low | **unclear** |
| Patel, 2016 | low | unclear | unclear | low | low | high | **high** |
| Pinket, 2016 | low | unclear | unclear | high | low | high | **high** |
| Rangelov, 2018 | low | low | unclear | low | low | high | **high** |
| Siega-Riz, 2011 | low | low | unclear | unclear | low | high | **high** |
| Smit, 2016 | unclear | unclear | unclear | low | low | high | **high** |
| Van der Gaar, 2014 | low | low | unclear | high | low | high | **high** |
| Story, 2012 | unclear | unclear | unclear | unclear | low | high | **high** |
| Veitch, 2011 | low | low | high | unclear | low | high | **high** |
| Vereecken, 2009 | unclear | unclear | unclear | low | high | high | **high** |
| Waters, 2017 | low | low | low | low | low | high | **high** |

| **Table S4: Risk of bias non-randomized controlled trials and non-controlled quasi-experimental studies** | | | | | | | | |
| --- | --- | --- | --- | --- | --- | --- | --- | --- |
| **Author, year** | **Bias due to confounding (age, sex, baseline)** | **Bias in selection of participants into the study** | **Bias in classification of interventions** | **Bias due to departures from intended interventions** | **Bias due to missing data** | **Bias in measurement of outcomes** | **Bias in selection of the reported result** | **Overall risk of bias** |
| **Abi Haidar, 2011** | serious | low | NA | NA | moderate | serious | moderate | **serious** |
| **Bea, 2015** | moderate | moderate | NA | NA | moderate | serious | moderate | **serious** |
| **Beets, 2014** | serious | moderate | NA | NA | unclear | moderate | moderate | **serious** |
| **Bender, 2013** | serious | serious | NA | NA | serious | serious | moderate | **serious** |
| **Brand, 2017** | moderate | serious | NA | NA | moderate | serious | moderate | **serious** |
| **Canavera, 2009** | serious | serious | NA | NA | unclear | serious | moderate | **serious** |
| **De Bourdeaudhuij, 2015** | moderate | moderate | low | serious | serious | serious | moderate | **serious** |
| **de Silva-Sanigorski, 2010** | moderate | moderate | low | unclear | critical | serious | moderate | **critical** |
| **Elder, 2014** | serious | moderate | low | moderate | serious | serious | moderate | **serious** |
| **Franks, 2017** | serious | serious | low | moderate | moderate | moderate | moderate | **serious** |
| **Freedman, 2010** | serious | critical | NA | serious | serious | serious | serious | **critical** |
| **Galvan, 2016** | moderate | low | NA | unclear | moderate | serious | moderate | **serious** |
| **Gittelson, 2010** | moderate | moderate | low | moderate | unclear | serious | serious | **serious** |
| **Hornsby, 2017** | moderate | moderate | NA | serious | unclear | serious | serious | **serious** |
| **Laurence, 2007** | serious | moderate | NA | unclear | serious | serious | moderate | **serious** |
| **Majumdar, 2013** | serious | moderate | low | moderate | serious | serious | moderate | **serious** |
| **McGarvey, 2004** | serious | moderate | low | moderate | serious | serious | moderate | **serious** |
| **Muckelbauer, 2009** | moderate | low | low | moderate | moderate | serious | moderate | **serious** |
| **Patel, 2011** | moderate | moderate | moderate | moderate | moderate | serious | moderate | **serious** |
| **Rauba, 2017** | serious | moderate | NA | unclear | low | serious | moderate | **serious** |
| **Romo, 2018** | serious | moderate | NA | unclear | moderate | serious | serious | **serious** |
| **Taylor, 2007** | moderate | moderate | low | unclear | serious | moderate | moderate | **serious** |
| **Wolfe, 2018** | serious | unclear | NA | unclear | unclear | serious | serious | **serious** |
| Abbreviation: NA=not applicable | | | | | | | | |

**Figure S1: Effect by focus of intervention**

**
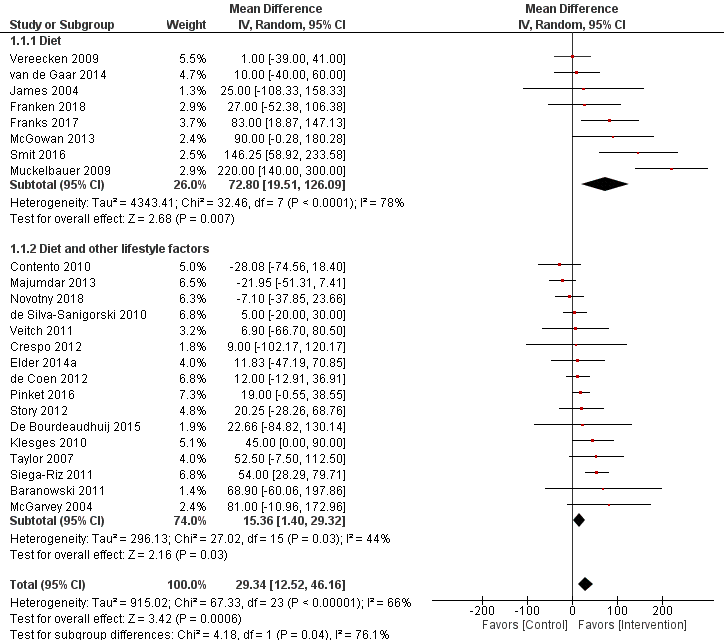
**

**Figure S2: Effect by intervention strategy**

**
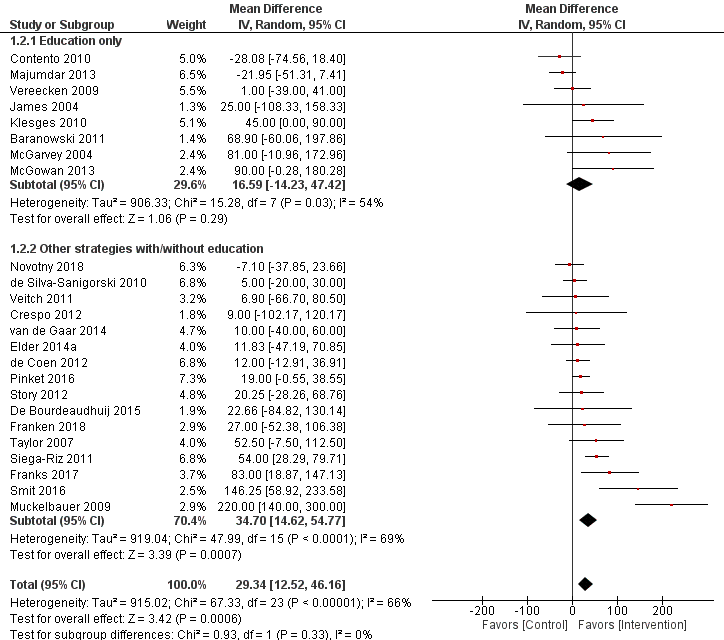
**

**Figure S3: Effect by intervention setting**

**
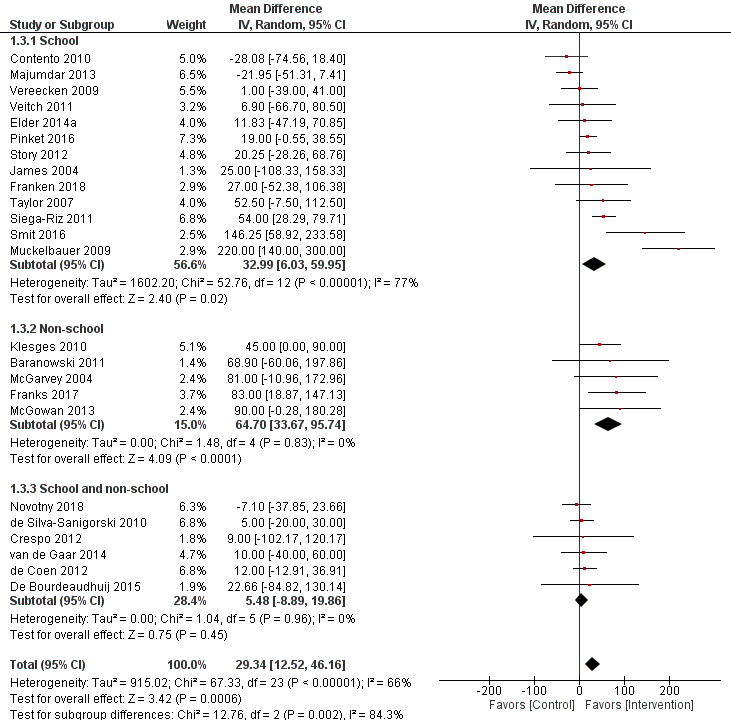
**

**Figure S4: Effect by socio-ecological level targeted**

**
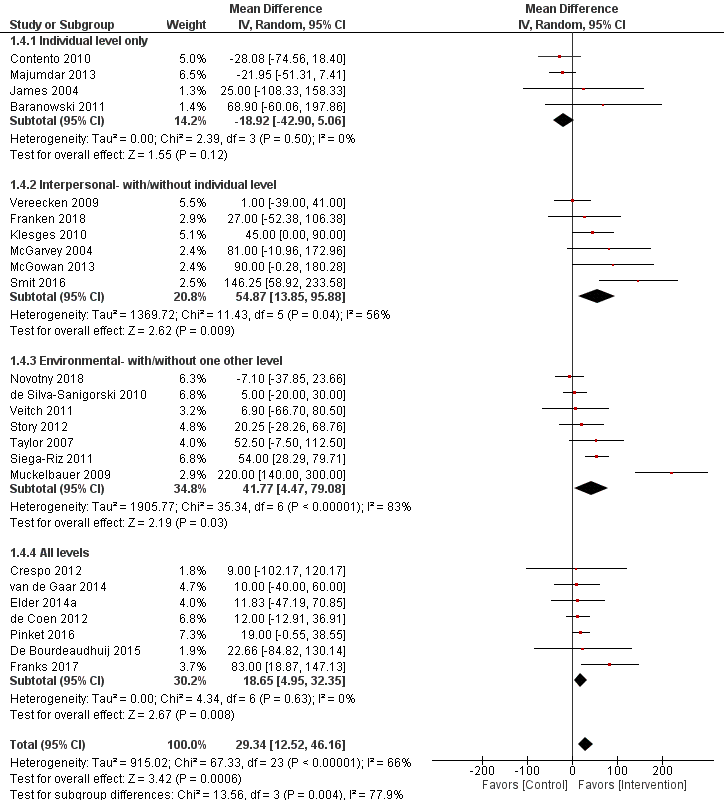
**

**Figure S5: Effect by mean age children**

**
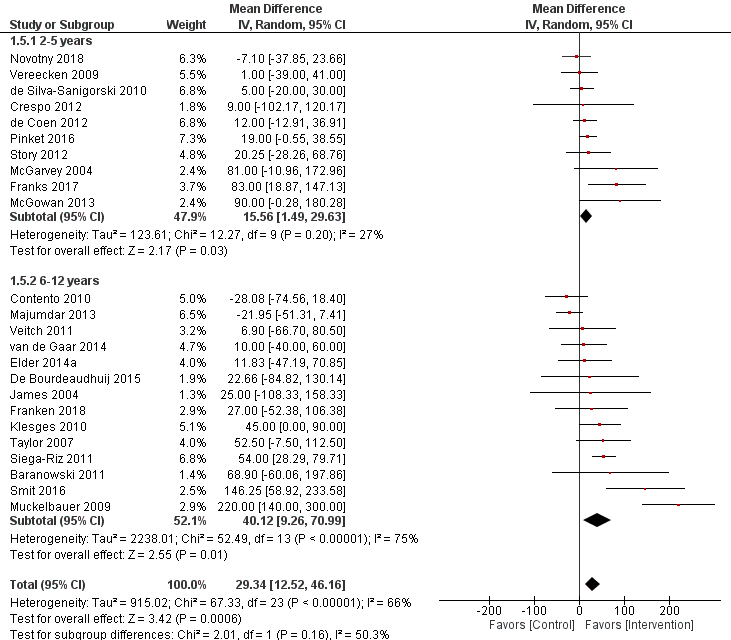
**

**Figure S6: Effect by study design**

**
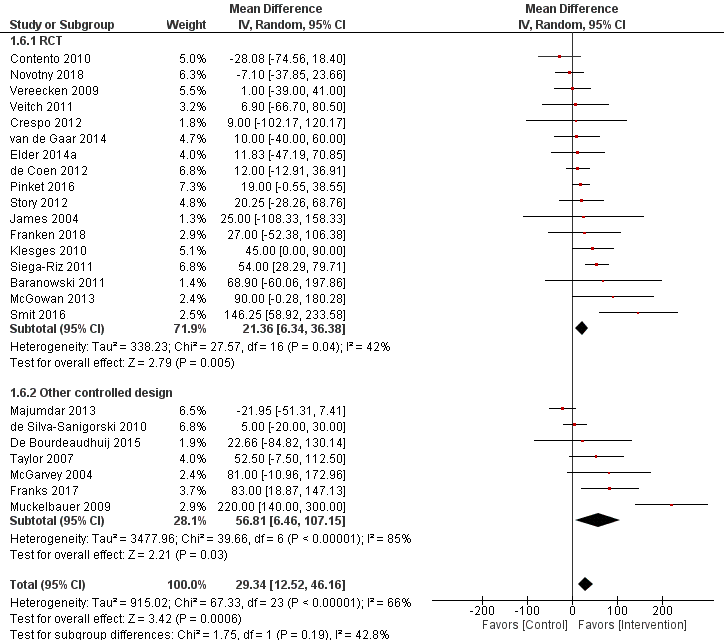
**

**Figure S7: Sensitivity analysis random-effects meta-analysis of the mean difference in children’s water consumption (in ml/day) between intervention and control group (N=24)**

**
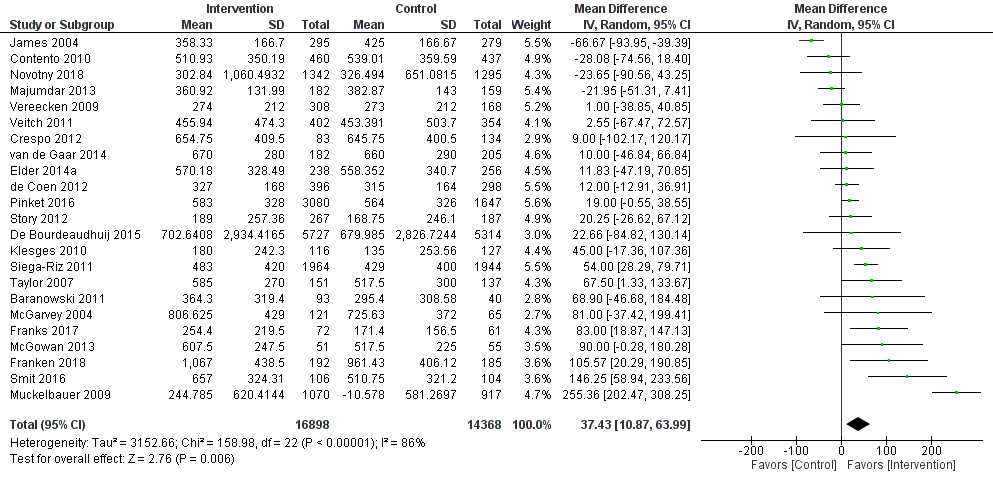
**

**Figure S8: Funnel plot of the mean difference (MD) in milliliter water consumption between intervention and control group against the standard error (SE) of the MD of all studies included in the meta-analysis**

**
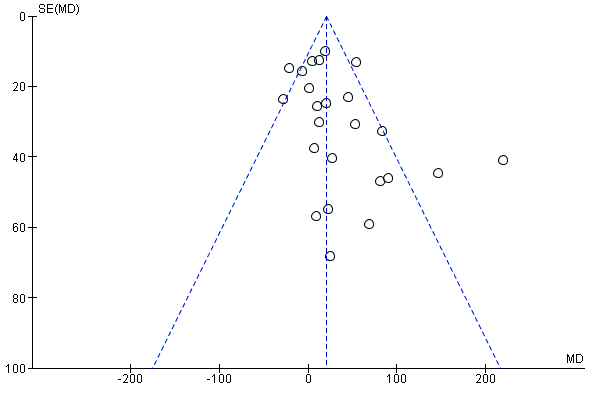
**
